# Variant in NHLRC2 leads to increased hnRNP C2 in developing neurons and the hippocampus of a mouse model of FINCA disease

**DOI:** 10.1186/s10020-020-00245-4

**Published:** 2020-12-09

**Authors:** Anniina E. Hiltunen, Salla M. Kangas, Steffen Ohlmeier, Ilkka Pietilä, Jori Hiltunen, Heikki Tanila, Colin McKerlie, Subashika Govindan, Hannu Tuominen, Riitta Kaarteenaho, Mikko Hallman, Johanna Uusimaa, Reetta Hinttala

**Affiliations:** 1 Medical Research Center Oulu and PEDEGO Research Unit, University of Oulu and Oulu University Hospital, PO Box 5000, 90014 Oulu, Finland; 2 Biocenter Oulu, University of Oulu, Oulu, Finland; 3 Proteomics Core Facility, Biocenter Oulu, Faculty of Biochemistry and Molecular Medicine, University of Oulu, PO Box 5400, Oulu, 90014 Finland; 4 Department of Immunology, Genetics and Pathology, Science for Life Laboratory, Uppsala University, Rudbeck Laboratory, Uppsala, Sweden; 5 A.I. Virtanen Institute, University of Eastern Finland, Kuopio, Finland; 6 The Hospital for Sick Children, Toronto, Canada; 7 Faculty of Medicine, University of Toronto, Toronto, Canada; 8 Tissue Engineering Laboratory, Hepia/HES-SO, University of Applied Sciences Western Switzerland, Geneva, Switzerland; 9 Department of Pathology, Cancer and Translational Medicine Research Unit, University of Oulu, Oulu, Finland; 10 Department of Pathology, Oulu University Hospital, Oulu, Finland; 11 Research Unit of Internal Medicine, Respiratory Research, University of Oulu, Oulu, Finland; 12 Medical Research Center Oulu and Unit of Internal Medicine and Respiratory Medicine, Oulu University Hospital, Oulu, Finland; 13 Clinic for Children and Adolescents, Paediatric Neurology Unit, Oulu University Hospital, Oulu, Finland

**Keywords:** FINCA, NHLRC2, hnRNP C1/C2, Crispr/Cas9, Neuronal precursor cell, 2D-DIGE

## Abstract

**Background:**

FINCA disease is a pediatric cerebropulmonary disease caused by variants in the NHL repeat-containing 2 (*NHLRC2*) gene. Neurological symptoms are among the first manifestations of FINCA disease, but the consequences of NHLRC2 deficiency in the central nervous system are currently unexplored.

**Methods:**

The orthologous mouse gene is essential for development, and its complete loss leads to early embryonic lethality. In the current study, we used CRISPR/Cas9 to generate an *Nhlrc2* knockin (KI) mouse line, harboring the FINCA patient missense mutation (c.442G > T, p.Asp148Tyr). A FINCA mouse model, resembling the compound heterozygote genotype of FINCA patients, was obtained by crossing the KI and *Nhlrc2* knockout mouse lines. To reveal NHLRC2-interacting proteins in developing neurons, we compared cortical neuronal precursor cells of E13.5 FINCA and wild-type mouse embryos by two-dimensional difference gel electrophoresis.

**Results:**

Despite the significant decrease in NHLRC2, the mice did not develop severe early onset multiorgan disease in either sex. We discovered 19 altered proteins in FINCA neuronal precursor cells; several of which are involved in vesicular transport pathways and actin dynamics which have been previously reported in other cell types including human to have an association with dysfunctional NHLRC2. Interestingly, isoform C2 of hnRNP C1/C2 was significantly increased in both developing neurons and the hippocampus of adult female FINCA mice, connecting NHLRC2 dysfunction with accumulation of RNA binding protein.

**Conclusions:**

We describe here the first NHLRC2-deficient mouse model to overcome embryonic lethality, enabling further studies on predisposing and causative mechanisms behind FINCA disease. Our novel findings suggest that disrupted RNA metabolism may contribute to the neurodegeneration observed in FINCA patients.

## Background

FINCA disease is a progressive cerebropulmonary disease (OMIM #618278) presenting with severe tissue fibrosis, neurodegeneration, and cerebral angiomatosis caused by pathogenic variants of the NHL Repeat Containing 2 (*NHLRC2*) gene (Uusimaa et al. 2018; Brodsky et al. 2020). Although FINCA patients have multi-organ manifestations including chronic hemolytic anemia, neurological symptoms were among the first to appear, by 2 months of age. Neuropathology of FINCA patients identified brain atrophy, vacuolar neurodegeneration, myelin loss with gliosis, cerebral angiomatosis, and neuronal depletion of the anterior horns of the spinal cord (Uusimaa et al. 2018). *NHLRC2* mRNA is present in several cell types and regions of the human and mouse brain (Zhang et al. 2014,2016). In addition, altered NHLRC2 and *NHLRC2* mRNA levels have been detected in neurodegenerative diseases such as Parkinson’s disease (PD) (Dijk et al. 2012) and Alzheimer’s disease (AD) (Long et al. 2016). A wide range of neural tube-related developmental malformations called developmental duplications have been reported in Angus cattle that are homozygous for p.Val311Ala substitution in the beta-propeller domain of NHLRC2 (Denholm 2017). Despite accumulating evidence for the importance of NHLRC2 in the central nervous system, its function in neurons is currently unknown.

NHLRC2 consists of an N-terminal thioredoxin (Trx)-like domain, a six-bladed β-propeller domain, and a C-terminal β-stranded region (Biterova et al. 2018). Structural analysis of the protein has revealed a highly conserved cleft between the Trx-like and β-propeller domains that forms a possible binding site for currently unknown substrates or interaction partners (Biterova et al. 2018). Recent in vitro studies have shed some light on the possible functions of NHLRC2. FINCA patient-derived and immortalized skin fibroblasts show enhanced differentiation to myofibroblasts and variants in NHLRC2 were found to affect the cytoskeleton organization and vesicle transport in normal human dermal fibroblasts (Paakkola et al. 2018). In macrophages, NHLRC2 was discovered to be a novel regulator of phagocytosis in two genome-wide knockout (KO) screens (Haney et al. 2018; Yeung et al. 2019), and has been proposed to affect phagocytosis via its effect on actin dynamics through RhoA–Rac1 signalling (Haney et al. 2018). In colon cancer cells, loss of NHLRC2 was found to increase the susceptibility of these cells to apoptosis induced by reactive oxygen species (ROS) (Nishi et al. 2017). However, the physiological function of NHLRC2 remains elusive.

The mouse ortholog of human NHLRC2 has 84% protein sequence similarity (Uusimaa et al. 2018), and it has been proposed that the function of NHLRC2 is conserved across species (Biterova et al. 2018). The complete loss of *Nhlrc2* leads to early embryonic lethality, highlighting its essential role in embryonic development (Uusimaa et al. 2018; Perez-Garcia et al. 2018; Delhotal 2016). X-gal staining has revealed widespread expression of *Nhlrc2* in a variety of organs during embryonic development (Uusimaa et al. 2018). *Nhlrc2* mRNA and *NHLRC2* mRNA expression is especially high during early brain development in mice and humans respectively (Uusimaa et al. 2018; Cardoso-Moreira et al. 2019; Miller et al. 2014), and in situ hybridization of embryonic day 14.5 mice shows high expression in the ventricular layer of the telencephalon (Diez-Roux et al. 2011). *Nhlrc2* has been detected in the transcriptional waves directing the differentiation of new born neurons in the neocortex (Telley et al. 2016). To date, molecular mechanisms related to *Nhlrc2* during brain development remain unexplored.

We describe here a novel FINCA knockin (KI) mouse line, generated by editing the mouse endogenous *Nhlrc2* gene to include the missense mutation identified in Finnish FINCA patients. This NHLRC2 deficient mouse escapes the embryonic lethality that has previously prevented further in vivo studies of a fully null KO mouse line. We compared the proteomes of FINCA and wild-type mouse embryonic neuronal precursor cells (NPCs), to elucidate the effect of NHLRC2 deficiency on developing neurons of the neocortex. The results obtained from our studies contribute to an understanding of the pathological mechanisms leading to neurodegeneration in FINCA disease.

## Materials and methods

A detailed description of the materials and methods is found in the Additional File 1. Unedited full images of the immunoblots are presented in Additional File 2.

## Results

### The first mouse model for FINCA has significantly decreased NHLRC2 protein levels but normal tissue histology

**Fig. 1:**
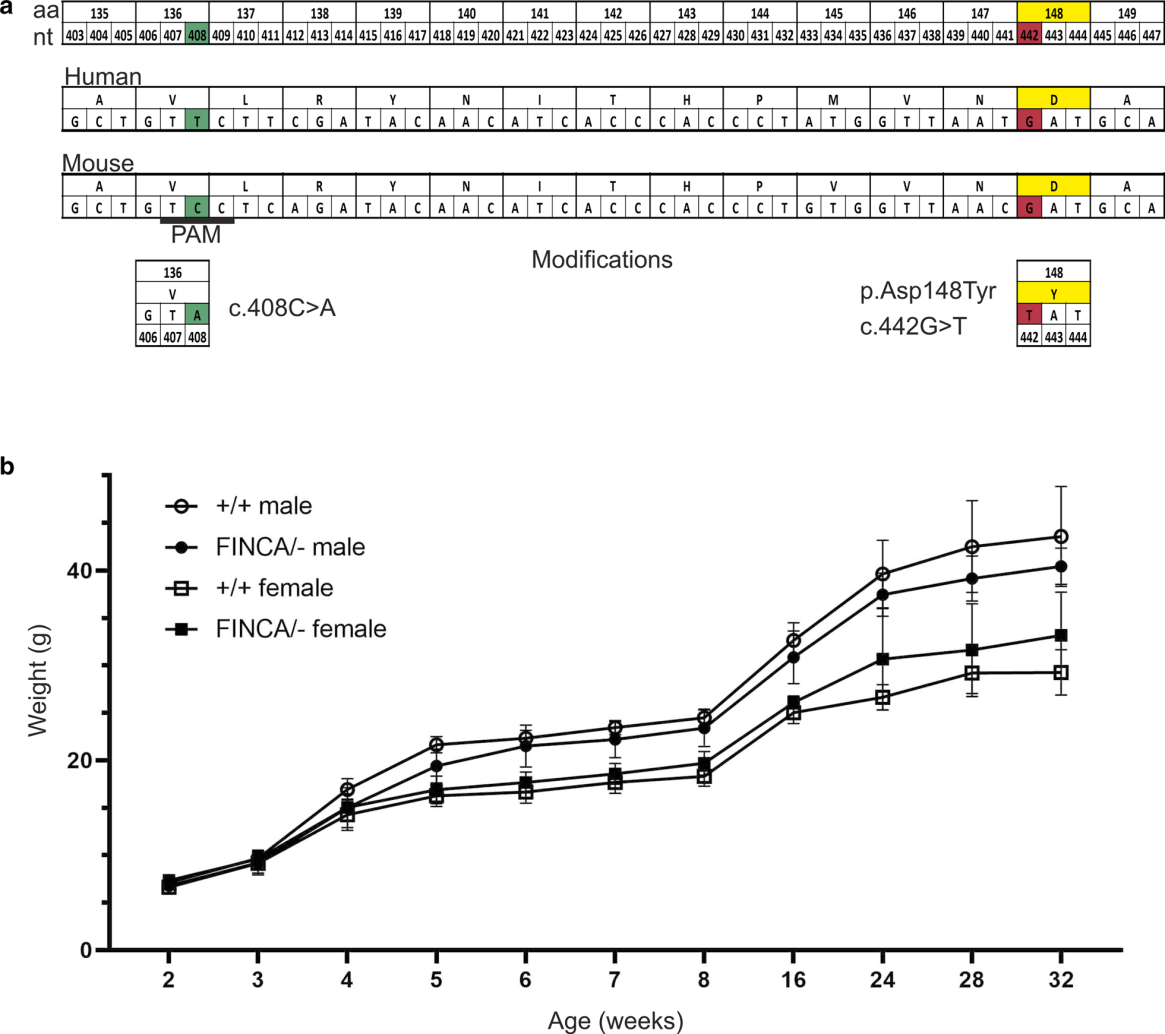
Schematic of the generated FINCA allele and body weight curve of *Nhlrc2*^FINCA/−^ and *Nhlrc2*^+/+^ mice. **a** Modified region of mouse and human amino acid (aa) and nucleotide sequence (nt) of the modified region. Silent mutation leading to removal of the PAM site and resulting in the addition of a TatI restriction site (green) was included in the ssODN. FINCA variant c.442G > T (red) and amino acid substitution p.Asp148Tyr (yellow) are shown. **b** Body weight curve showing no significant difference in weight gain for either sex between *Nhlrc2*^FINCA/−^ and *Nhlrc2*^+/+^ mice during the 32- week observation period (two-way ANOVA). *Nhlrc2*^FINCA/−^ male (N = 6), *Nhlrc2*^+/+^ male (N = 6) *Nhlrc2*^FINCA/−^ female (N = 6) *Nhlrc2*^+/+^ female (N = 5). Mean and standard deviation (SD) shown

FINCA disease is caused by pathogenic variants in *Nhlrc2*. The mutation site and its adjacent area are highly conserved between human and mouse (Fig. 1a). We generated a mouse line with the FINCA patient variant c.442G > T by editing the mouse endogenous *Nhlrc2* gene using the CRISPR/Cas9 method (Cong et al. 2013; Mali et al. 2013; Inui et al. 2014) (Fig. 1a, Additional File 1: Fig. S1). The heterozygous FINCA (hereafter *Nhlrc2*^FINCA/+^) and heterozygous *Nhlrc2* KO, C57BL/6N-A^*tm1Brd*^Nhlrc2^*tm1a(KOMP)Wtsi*^/WtsiOulu (hereafter *Nhlrc2*^−/+^) (Skarnes et al. 2011) mice were crossed to obtain mice (*Nhlrc2*^FINCA/−^) mimicking the genotype of compound heterozygote FINCA patients with the missense variant and a frameshifting nonsense variant on the other allele (Uusimaa et al. 2018).

**Fig. 2:**
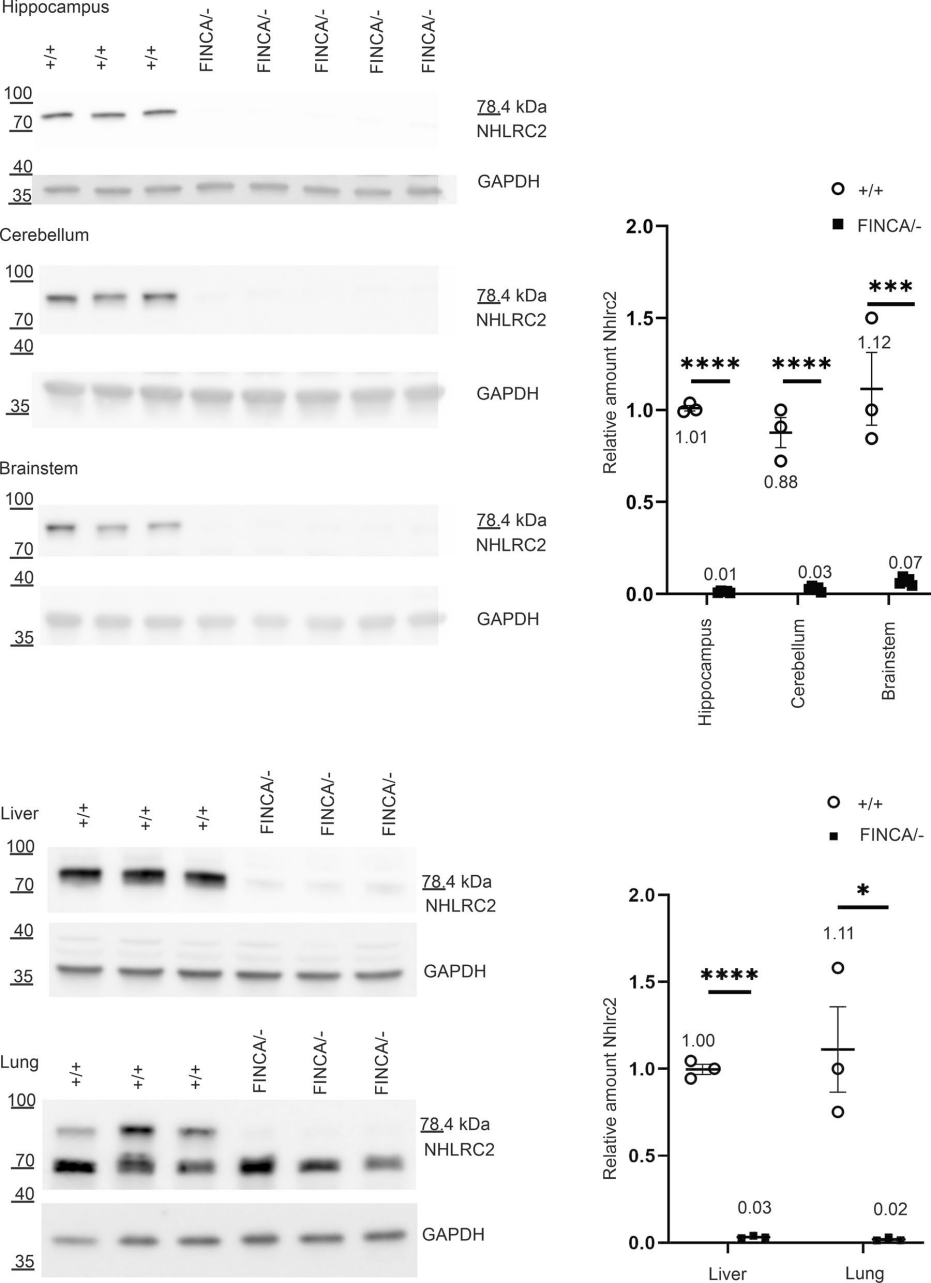
NHLRC2 is significantly decreased in *Nhlrc2*^FINCA/−^ mouse tissues. SDS-PAGE immunoblotting showing NHLRC2 in hippocampus, cerebellum, and brainstem of 13-week-old and lung and liver of 32-week-old *Nhlrc2*^FINCA/−^ and *Nhlrc2*^+/+^ mouse tissue lysates. The amount of NHLRC2 decreased to 1.1% in the hippocampus (p < 0.0001), 3.3% in the cerebellum (p < 0.0001), 6.2% (p = 0.0004) in the brainstem, 3.2% (p < 0.0001) in the liver, and 1.7% (p = 0.011) in the lung of Nhlrc2 ^FINCA/−^ mice compared to wild-type mice (Student’s t-test). The expected size of full length NHLRC2 is 78.4 kDa. Protein amounts are relative to one of the wild-type samples, and GAPDH was used for normalization. Dot blots show individual data points, group mean and standard error of the mean (SEM). ****p < 0.0001, ***p < 0.001

Immunoblotting with NHLRC2 antibody recognized a band corresponding to the predicted size of mouse NHLRC2 (78.43 kDa, Uniprot.org, 25.3.20), showing a consistent decrease in all *Nhlrc2*^FINCA/−^ mouse tissues evaluated (Fig. 1, Additional File 1: S2). As in humans, NHLRC2 protein was present in all analysed mouse tissue homogenates (Additional File 1: Fig. S2). Compound heterozygous *Nhlrc2*^FINCA/−^ mice had a more prominent decrease in the amount of NHLRC2 compared to homozygous *Nhlrc2*^FINCA/FINCA^ mice (Additional File 1: Fig. S3). We selected a set of tissues affected by FINCA disease (hippocampus, cerebellum, brainstem, lung, liver) and determined the amount of NHLRC2 protein in the *Nhlrc2*^FINCA/−^ mice. NHLRC2 was decreased to 1.1% in the hippocampus (p < 0.0001), to 3.3% (p < 0.0001) in the cerebellum, and to 6.2% (p = 0.0004) in the brainstem of *Nhlrc2*^FINCA/−^ mice compared to wild-type mice (Fig. 2). *Nhlrc2*^FINCA/−^ mouse lung and liver lysates had 1.7% (p = 0.011) and 3.2% (p < 0.0001) of residual NHLRC2, respectively, compared to *Nhlrc2*^+/+^ mice (Fig. 2).

**Fig. 3:**
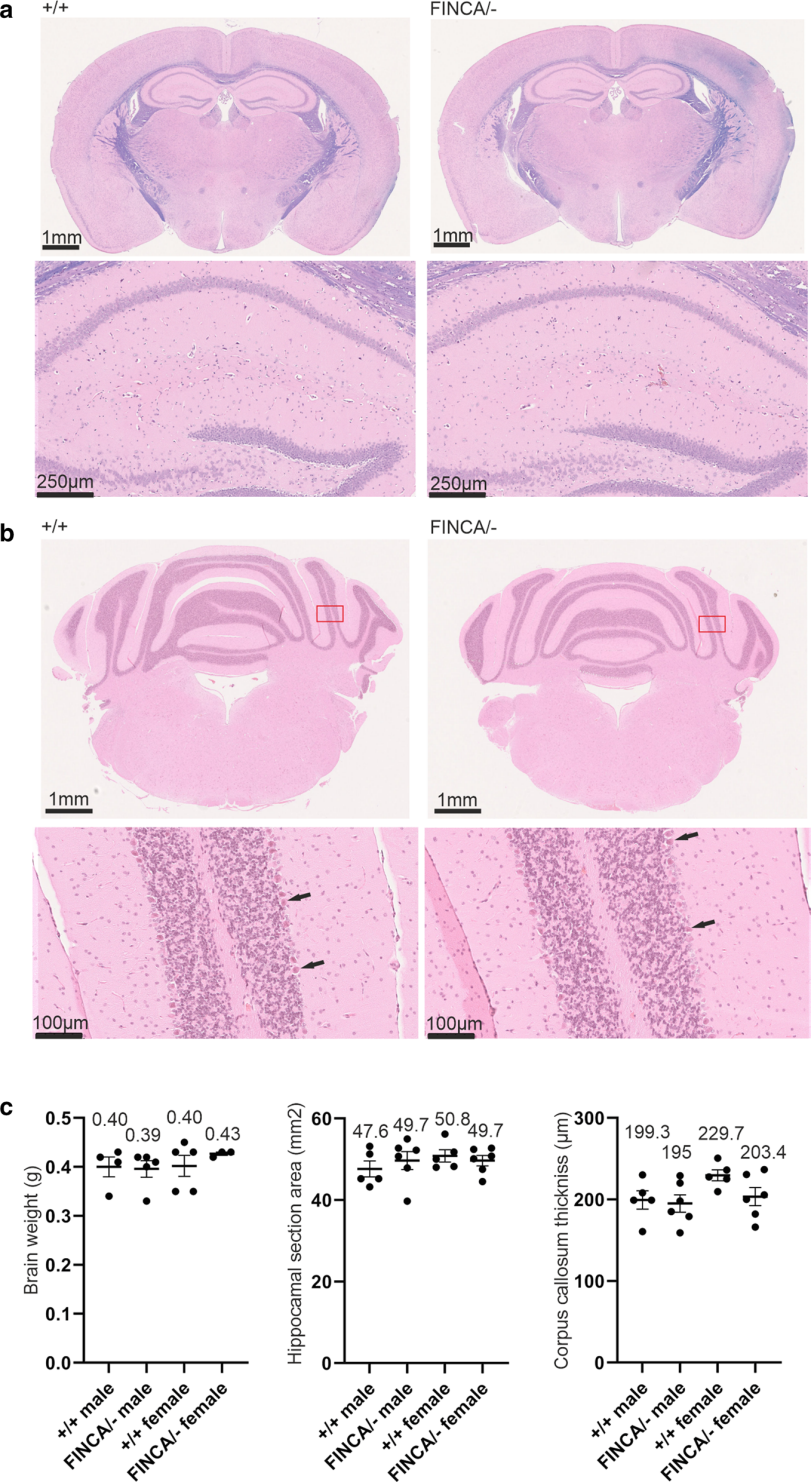
*Nhlrc2*^FINCA/−^ mice showed no morphological changes, neurodegeneration, or demyelination in the brain. **a** Upper: Representative image of 32-week-old female *Nhlrc2*^FINCA/−^ and *Nhlrc2*^+/+^ hippocampal section stained with Luxol fast blue. Lower: a higher magnification showed no neurodegeneration in the dorsal hippocampus. **b** Upper: Representative image of 32-week-old male *Nhlrc2*^FINCA/−^ and *Nhlrc2*^+/+^ cerebella. Lower: Higher magnification of the area inside the red box showing normal granule and Purkinje cells (black arrow). **c** Dot blots showing brain weight after fixation; area of the hippocampal section and thickness of corpus callosum measured from the midline. Individual datapoints, means and SEM are shown. There were no significant changes (Student’s t-test)

Compound heterozygous *Nhlrc2*^FINCA/−^ mice (6 males and 6 females) and their wild-type *Nhlrc2*^+/+^ litter mates (6 males and 5 females) were observed for 32 weeks. *Nhlrc2* ^FINCA/−^ mice appeared normal at birth, gained weight comparably to wild-type litter mates (Fig. 1b), and reproduced normally (Additional File 1: Table S3). *Nhlrc2*^FINCA/−^ mice did not develop clinical signs during the observation period, and they were euthanized for tissue-based evaluation of FINCA disease-like phenotype. There was no brain atrophy or abnormal histology identified in hippocampal and cerebellar sections of *Nhlrc2*^FINCA/−^ mice (Fig. 3). Lung and liver fibrosis and hemolytic anemia are also common manifestations of FINCA disease (Uusimaa et al. 2018; Brodsky et al. 2020). There were no observable abnormalities in lung and liver sections of *Nhlrc2*^FINCA/−^ mice (Additional File 1: Fig. S4), nor were there significant changes in hemoglobin or hematocrit values (Additional File 1: Table S4).

Although *Nhlrc2*^FINCA/−^ mice did not recapitulate the tissue manifestations of FINCA disease, the decrease in NHLRC2 protein at the tissue level is striking. Thus, our *Nhlrc2*^FINCA/−^ mouse model enables studies of the changes at the molecular and cellular level resulting from decreased NHLRC2. This may reveal the affected pathways that predispose and ultimately lead to a severe disease phenotype.

### Characterization of *Nhlrc2*^FINCA/−^ embryonic NPCs

**Fig. 4:**
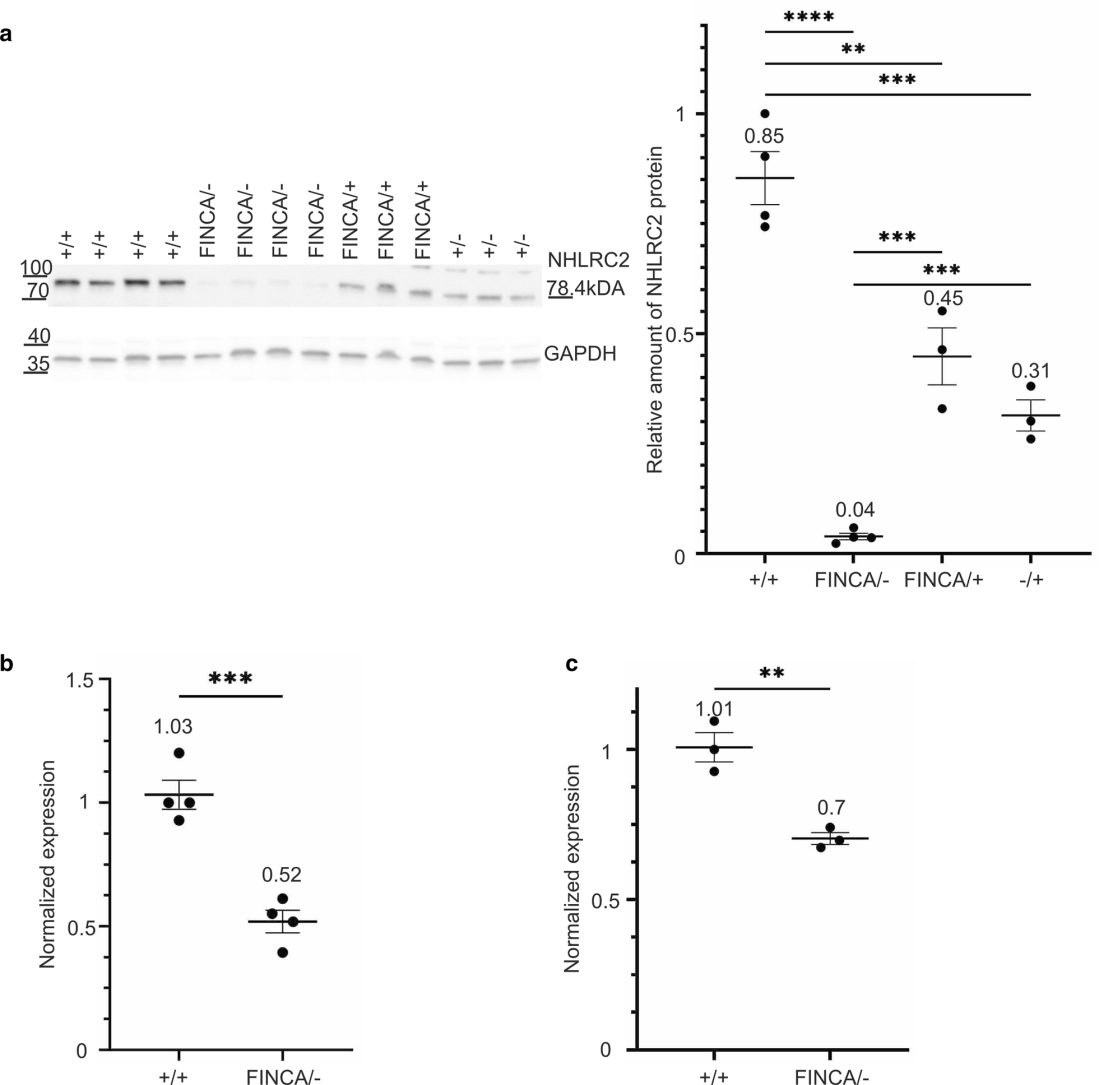
*Nhlrc2*^FINCA/−^ mouse NPCs display a significant decrease in *Nhlrc2* expression and in the amount of NHLRC2. **a** Immunoblotting and scatter plot of band intensities of NHLRC2 in four *Nhlrc*2^+/+^, four compound heterozygous *Nhlrc2*^FINCA/−^, three heterozygous *Nhlrc2*^FINCA/+^, and three heterozygous Nhlrc2^+/− ^ NPC whole-cell lysates. *Nhlrc2*^FINCA/−^ (5%, p < 0.0001), *Nhlrc2*^FINCA/+^ (53%, p = 0.0062), and *Nhlrc2*^+/−^ (37%, p = 0.0009) had significantly decreased NHLRC2 levels compared to wild-type NPCs. *Nhlrc2*^FINCA/−^ also differed significantly from *Nhlrc2*^FINCA/+^ (p = 0.0007) and *Nhlrc2*^+/−^ (p = 0.039) (Student’s t-test). Protein amounts are relative to one of the wild-type samples and GAPDH was used for normalization. **b** qPCR from four *Nhlrc2*^FINCA/−^ and four *Nhlrc2*^+/+^ showed 50.2% mRNA expression (p = 0.0005) in the *Nhlrc2*^FINCA/−^ NPCs when using primers designed over the intron between exons 4 and 5 of *Nhlrc2,* where the KO first allele construct resides and terminates the transcription. **c** qPCR, using a primer pair over the intron between exons 3 and 4 of *Nhlrc2*, from three *Nhlrc2*^FINCA/−^ and three *Nhlrc2*^+/+^ NPCs, showed a significant decrease in *Nhlrc2*^FINCA\−^ (70%, p = 0.0044) cells compared to *Nhlrc2*^+\+^ (Student’s t-test). Scatter plots show individual data points, group means, and SEM. ****p < 0.0001, ***p < 0.001, **p < 0.01, *p < 0.05

To study developing neurons, we established cortical NPC cultures from E13.5 embryos from heterozygous *Nhlrc2*^FINCA/+^ and *Nhlrc2*^−/+^ matings. *Nhlrc2*^FINCA/−^ embryos appeared indistinguishable from wild-type littermates, and the NPCs grew normally in vitro without any apparent phenotype (Additional File 1: Fig. S5). The amount of NHLRC2 was decreased to 5% (p < 0.0001) in *Nhlrc2*^FINCA/−^ NPCs compared to wild-type cells (Fig. 4a). Heterozygosity in either the FINCA or KO allele led to a significant change in protein level, to 53% (p = 0.0062) and 37% (p = 0.0009), respectively (Fig. 4a).

The *LacZ* cassette of the KO allele, *Nhlrc2*^*tm1a(KOMP)Wtsi*^, resides in the intron between exons 4 and 5 of *Nhlrc2,* where it leads to the termination of transcription. Since KO first conditional-ready tma1 alleles can skip over the *LacZ* cassette and restore the original gene expression to some extent (White et al. 2013), we compared the expression prior to and after the *LacZ* site by quantitative polymerase chain reaction (qPCR) using primers preceding and primers overlapping the intronic area with the cassette. qPCR preceding the cassette resulted in 70% *Nhlrc2* mRNA in *Nhlrc2*^FINCA/−^ NPCs compared to expression levels in wild-type NPCs (p = 0.004) (Fig. 4c). The expression of the full-length *Nhlrc2* mRNA was 50.2% in the *Nhlrc2*^FINCA/−^ NPCs compared to wild-type NPCs (p = 0.0005) (Fig. 4b). Similarly, the full-length *Nhlrc2* mRNA was decreased to 50% in immortalized FINCA patient fibroblast expressing only the missense variant (Uusimaa et al. 2018). Collectively, this suggests that *Nhlrc2* mRNA encoding p.Asp148Tyr is stable both in humans and in mice. The KO allele of *Nhlrc2*^*tm1a(KOMP)Wtsi*^ retains 20% expression of the truncated protein with LacZ without a sign of leakage of the full-length *Nhlrc2*.

### 2D-DIGE revealed 19 proteins affected by *Nhlrc2*^FINCA/−^ genotype in embryonic cortical NPCs

To further study the effect of NHLRC2 deficiency on developing neurons, we compared *Nhlrc2*^FINCA/−^ and *Nhlrc2*^+/+^ NPCs with two-dimensional difference gel electrophoresis (2D-DIGE). 2D-DIGE revealed 21 spots with significantly changed intensities between the two genotypes (Additional File 1: Fig. S6). Further analysis using mass spectrometry (MS) identified 19 unique proteins (Table 1, Additional File 1: Table S5). Among them, only the transitional endoplasmic reticulum ATPase (VCP) was decreased in *Nhlrc2*^FINCA/−^ NPCs, whereas all other proteins were increased in the mutant in comparison to *Nhlrc2*^+/+^ NPCs.

**Table 1:** List of significantly changed proteins from 2D-DIGE identified by MS (p < 0.05, minimum 1.5-fold change)

Spot	Protein	Description	Ratio	t-Test
1	VCP	Transitional endoplasmic reticulum ATPase	− 2.07	0.01
2	ANXA6	Annexin A6	1.85	0.04
3	PLS3	Plastin-3	1.62	0.05
4	SNX6	Sorting nexin-6	1.62	0.02
5	hnRNP C1/C2	Heterogeneous nuclear ribonucleoprotein C1/C2 (isoform C2)	1.54	0.03
6	UBL7	Ubiquitin-like protein 7	1.95	0.04
7	GPD1L	Glycerol-3-phosphate dehydrogenase 1-like protein (isoform 1 or 2)	1.54	0.03
8	TIMM29	Mitochondrial import inner membrane translocase subunit Tim29	1.57	0.03
9	UBA1	Ubiquitin-like modifier-activating enzyme 1 (C-terminal fragment)	2.45	0.04
10	PRDX6	Peroxiredoxin-6	1.90	0.01
11	PSMA2	Proteasome subunit α type-2	1.66	0.04
12	FTL1	Ferritin light chain 1	1.77	0.04
13	RBM8A	RNA-binding protein 8A (isoform 1)	1.60	0.05
14	FTL1	Ferritin light chain 1 (N-terminal fragment)	2.14	0.02
15	PFDN2	Prefoldin subunit 2	1.68	0.05
16	HIST1H4A	Histone H4	1.62	0.03
17	HIST1H4A	Histone H4	2.89	0.03
18	DYNLRB1	Dynein light chain roadblock-type 1	1.68	0.04
19	ABRACL	Costars family protein ABRACL	1.63	0.04
20	PEA15	Astrocytic phosphoprotein PEA-15 (isoform 1)	2.04	0.04
21	BRK1	Protein BRICK1	1.81	0.03

The ratio indicates the change of the normalized spot volumes in *Nhlrc2*^FINCA/−^ in comparison to *Nhlrc2*^+/+^ NPCs

### STRING protein–protein interaction analysis

Protein–protein interaction analysis of the changed mouse proteins observed in 2DE-DIGE, and their human orthologs, showed significant enrichment of interaction for the identified proteins (mouse p = 0.00056 and human p = 0.00204) (Additional File 1: Fig. S7). Interestingly, VCP was found to be a central node in both networks; however, our further qPCR, SDS-PAGE, and immunoblotting experiments failed to show significant differences between *Nhlrc2*^FINCA/−^ and *Nhlrc2*^+/+^ NPCs (Additional File 1: Table S6, Fig. S8). Collectively, this suggests that the observed change for VCP is the result of an additional posttranslational modification which, in turn, might hinder detection with the antibodies.

### NHLRC2 affects proteins enriched in vesicular compartments

Gene ontology (GO) enrichment analysis of human orthologs for cellular components revealed that the majority of changed proteins were linked to extracellular exosomes, vesicles, and protein-containing complexes, as well as cytosol (Table 2). No enrichment was found in certain molecular functions or biological processes in the GO term analysis. Similarly, NHLRC2 has previously been linked to an exceptionally large variety of biological processes (Paakkola et al. 2018; Haney et al. 2018; Yeung et al. 2019).

**Table 2:** Gene ontology enrichment analysis of human orthologs of the identified genes

GO cellular component complete	*Homo sapiens* REFLIST (20,996)	Observed (19)	Expected	Fold enrichment	±	Raw p-value	FDR
Azurophil granule lumen (GO:0035578)	90	3	0.08	36.84	+	7.72E−05	1.94E−02
→ Azurophil granule (GO:0042582)	154	3	0.14	21.53	+	3.63E−04	4.86E−02
→ Vesicle (GO:0031982)	3868	11	3.5	3.14	+	1.45E−04	2.92E−02
Primary lysosome (GO:0005766)	154	3	0.14	21.53	+	3.63E−04	4.56E−02
→ Lysosome (GO:0005764)	708	6	0.65	9.3	+	2.91E−05	9.73E−03
→ Lytic vacuole (GO:0000323)	708	6	0.65	9.3	+	2.91E−05	1.17E−02
→ Vacuole (GO:0005773)	809	6	0.74	8.14	+	6.10E−05	1.75E−02
Secretory granule lumen (GO:0034774)	320	4	0.29	13.81	+	1.79E−04	3.27E−02
→ Cytoplasmic vesicle lumen (GO:0060205)	324	4	0.29	13.64	+	1.88E−04	3.14E−02
→ Vesicle lumen (GO:0031983)	326	4	0.3	13.56	+	1.92E−04	2.97E−02
Extracellular exosome (GO:0070062)	2098	10	1.9	5.27	+	3.98E−06	7.99E−03
→ Extracellular vesicle (GO:1903561)	2119	10	1.92	5.21	+	4.36E−06	4.37E−03
→ Extracellular organelle (GO:0043230)	2124	10	1.92	5.2	+	4.45E−06	2.98E−03
→ Extracellular space (GO:0005615)	3349	10	3.03	3.3	+	2.45E−04	3.51E−02
Cytosol (GO:0005829)	5229	14	4.76	2.94	+	1.21E−05	6.08E−03
Protein-containing complex (GO:0032991)	5520	13	5.03	2.58	+	1.59E−04	3.55E−02

Presented hierarchically with the most specific subclass first, with its parent terms directly below it. Related classes in an ontology are grouped. Results shown for false discovery rate (FDR) < 0.05. (geneontology.org, 22.5.20)

### Heterogeneous nuclear ribonucleoprotein C1/C2 is increased in NPCs and in the hippocampus of adult *Nhlrc2*^FINCA/−^ mice

**Fig. 5:**
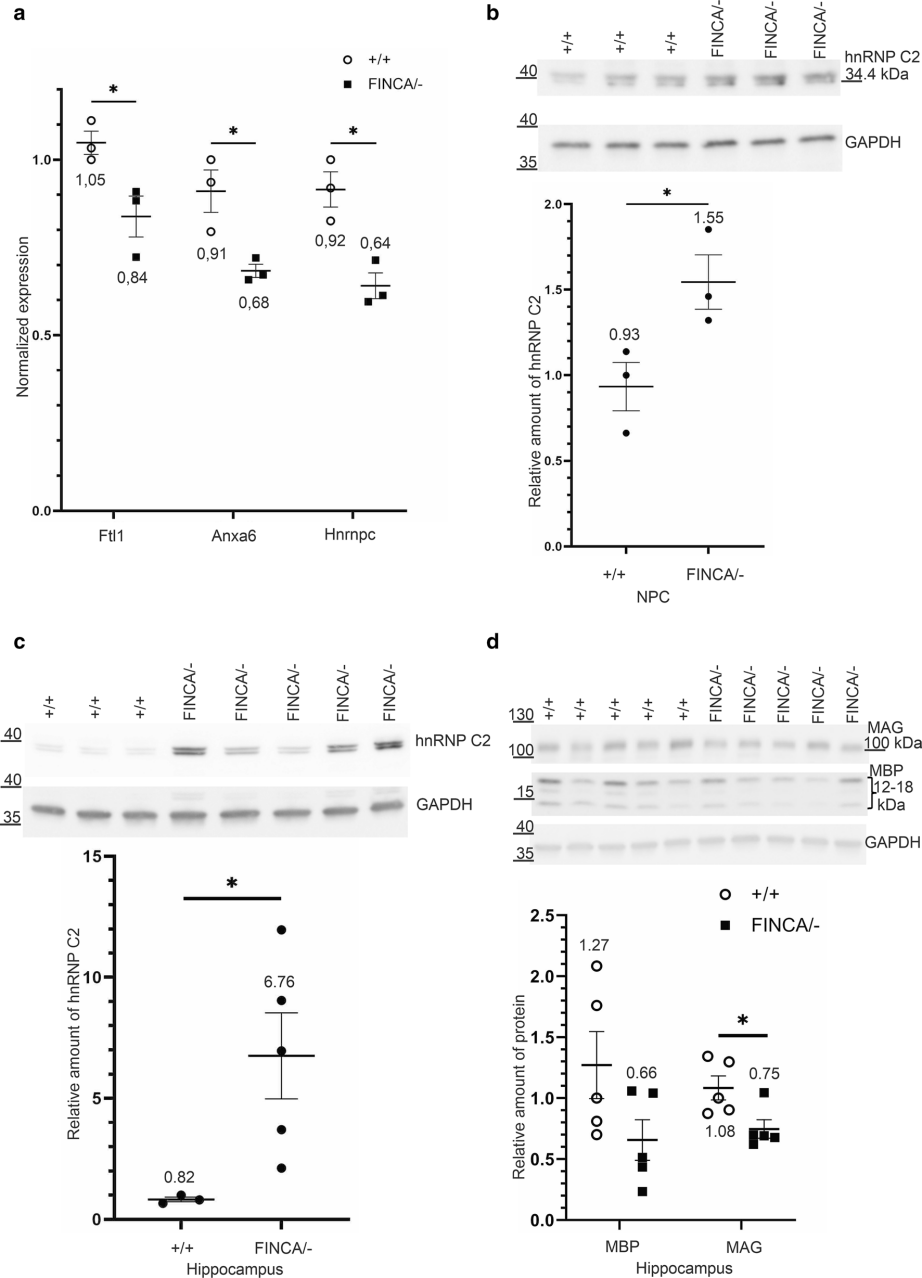
qPCR and immunoblotting showed changes in the hnRNPC C2 level in NPCs and hippocampus of *Nhlrc2*^FINCA/−^ mouse. **a** qPCR results of the three protein transcripts; *Ftl1*, *Anxa6*, and *Hnrnpc,* showed significantly changed RNA levels in *Nhlrc2*^FINCA/−^ (square) compared to *Nhlrc2*^+/+^ (round) NPCs, 80% (p = 0.035), 75%, (p = 0.023), and 70% (p = 0.012) respectively (Student’s t-test). Expression was normalized against one wild-type sample. Individual data points, group mean, and SEM are shown. **b** SDS-PAGE immunoblot and measured protein levels of three *Nhlrc2* ^−/−^ and *Nhlrc2*^FINCA/−^ NPC lysates revealed increased hnRNP C2 (165% p = 0.045). **c** SDS-PAGE immunoblot and measured protein levels from 13-week-old female *Nhlrc2*^FINCA/−^ and wild-type hippocampal tissue lysates showed increased hnRNP C2 (820%, p = 0.046). hnRNP C2-specific antibody was used and the expected size of hnRNP C2 is 34.4 kDa. **d** SDS-PAGE immunoblot and measured protein levels from 5-week-old male *Nhlrc2*^FINCA/−^ and wild-type hippocampal tissue lysates for myelin basic protein (MBP) and myelin associated glycoprotein (MAG). MAG was significantly decreased in Nhlrc2^FINCA/−^ (69%, p = 0,027). Protein amounts are relative to one of the wild-type samples and GAPDH was used for normalization. Individual data points, group mean, and SEM are shown. Statistical analysis was done using Student's t-test *p < 0.05

We next performed qPCR to study whether the changes observed in 2D-DIGE could be explained by changes in transcription. Only three of the identified proteins, annexin A6 (ANXA6), ferritin light chain 1 (FTL1), and heterogeneous nuclear ribonucleoproteins C1/C2 (hnRNP C1/C2), had significantly changed transcriptional levels in *Nhlrc2*^FINCA/−^ NPCs (Fig. 5a and Additional File 1: Table S6). Contrary to the increased intensity observed in 2D-DIGE, the expression of these genes was decreased. *Anxa6* had 75% (p = 0.023), *Ftl1* 80% (p = 0.035), and *Hnrnpc* 70% (p = 0.012) expression in mutant NPCs compared to the wild-type NPCs. Hence, the changes in 2D-DIGE are likely to be differences in the protein turnover rates or in the chemical and physical properties of the proteins, caused by, for example, changes in their post-translational modifications (PTMs), rather than differences in their expression.

HnRNP C1/C2, isoform C2 showed elevated protein levels in *Nhlrc2*^FINCA/−^ compared to *Nhlrc2*^+/+^ NPCs in the 2D-DIGE analysis (Table 1), whereas qPCR indicated decreased mRNA levels (Fig. 5a and Additional File 1: Table S6). SDS-PAGE and immunoblotting confirmed the observed changes at the protein level with a 65% increase in *Nhlrc2*^FINCA/−^ NPCs (p = 0.045) (Fig. 5b). Immunocytochemistry (ICC) staining indicated normal localization of hnRNP C1/C2 in both mutant and wild-type cells (Additional File 1: Fig. S9).

**Fig. 6:**
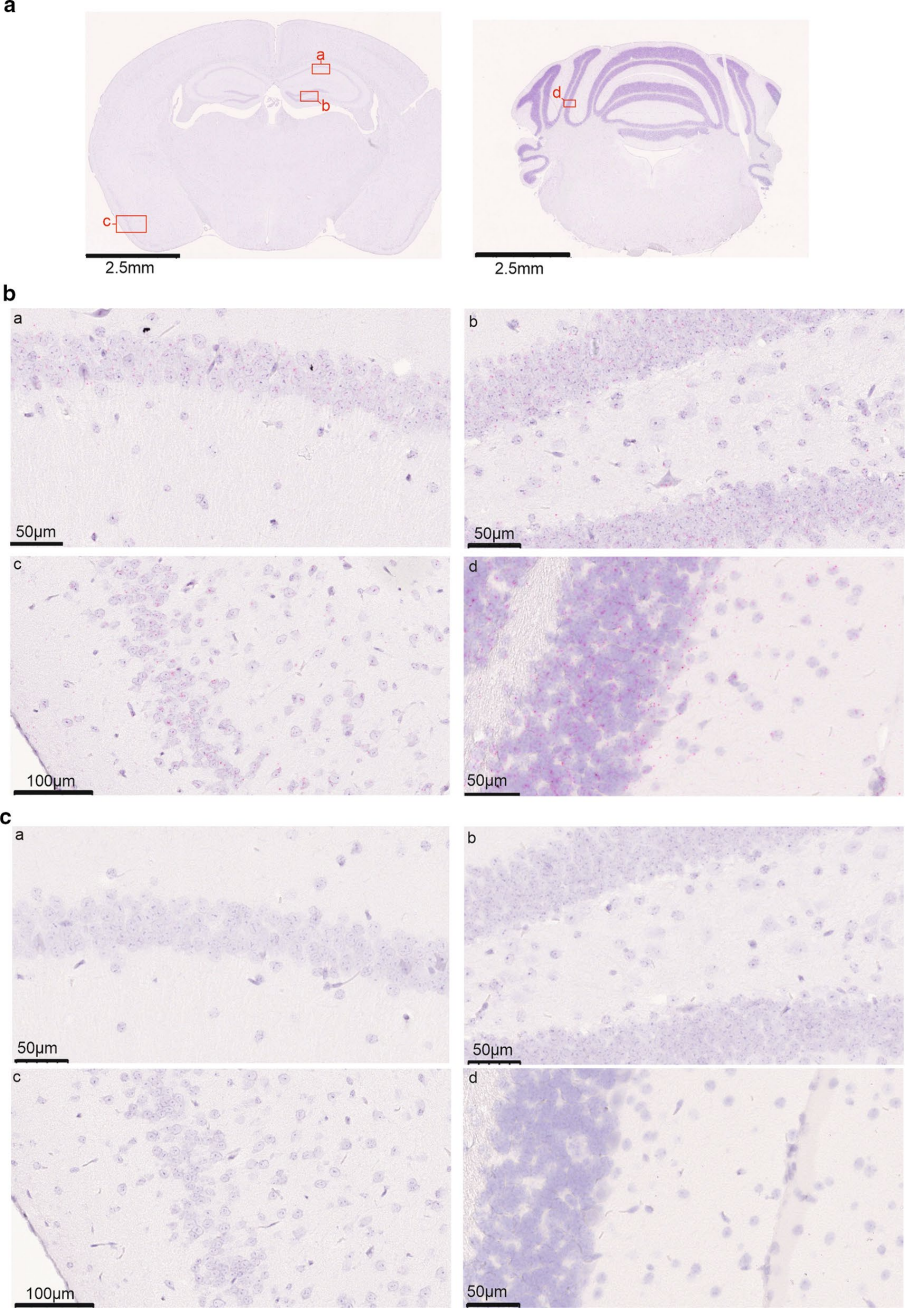
Representative in situ hybridization of *Nhlrc2* in the brain of a 32-week-old male C57BL/6NCrl mouse. **a** Overview image of ISH with *Nhlrc2* probe (red). Red boxes mark the magnified areas: a CA1 layer of hippocampus, b dentate gyrus of hippocampus, c piriform cortex, d cerebellar hemisphere. **b** *Nhlrc2* expression in areas a–d. A strong signal was observed from the pyramidal cell layer of the hippocampus and piriform cortex as well as dentate and cerebellar granule cells. **c** Negative control from areas a–d

In situ hybridization (ISH) of *Nhlrc2* showed ubiquitous expression throughout the adult brain of 32-week-old male mice, with the most prominent expression in cerebellar granule cells, followed by granule cells in the dentate gyrus, and then by pyramidal cells in hippocampal CA1 layer and layer 2 of the piriform cortex (Fig. 6). ISH of *Nhlrc2*^FINCA/−^ brain revealed a similar expression pattern of the mutated *Nhlrc2* mRNA to that of the wild-type (Additional File 1: Fig. S11). Since the hippocampus, cerebellum, and brainstem are affected in FINCA disease, we performed SDS-PAGE and immunoblotting analyses of hnRNP C2 in these tissues from 13-week-old female *Nhlrc2*^FINCA/−^ and *Nhlrc2*^+/+^ mice. Interestingly, hnRNP C2 (820%, p = 0.046) was observed to be significantly increased in the hippocampus of *Nhlrc2*^FINCA/−^ in comparison to *Nhlrc2*^+/+^ (Fig. 5c), but not in the cerebellum or brainstem of the same animals (Additional File 1: Fig. S10). Furthermore, hnRNP C2 has been found to affect transcription of myelination related genes in human neuroblastoma cells (Iwata et al. 2011). Immunoblotting of 5-week-old mouse hippocampus lysates revealed a significant decrease in the amount of myelin-associated glycoprotein (MAG) in Nhlrc2^FINCA/−^ mice compared to wild type mice (69%, p = 0.027) (Fig. 5d). Myelin basic protein (MBP) also showed a decrease in the amount of protein but was not statistically significant (52%, p = 0.092) (Fig. 5d). These findings associate hnRNP C2 with the FINCA disease pathology and suggest that NHLRC2 plays an important role especially in the hippocampus.

## Discussion

KI mouse models can be considered precision disease models that recapitulate the single pathogenic variant created into the endogenous target gene, omitting any artificial overexpression of gene products seen in conventional transgenic mouse models. Here, we generated a KI mouse line with the FINCA patient variant c. 442G < T, p.Asp148Tyr, in the endogenous mouse *Nhlrc2* gene. By crossing this mouse line with the *Nhlrc2* KO mouse line, we produced a similar compound heterozygous genotype to that identified in FINCA patients. Compound heterozygous *Nhlrc2*^FINCA/−^ mice overcame the embryonic lethality of *Nhlrc2* KO mice and developed normally in utero. Since the missense mutation does not reside in the most evolutionarily conserved area of *NHLRC2* that is predicted to be the active core of the protein (Biterova et al. 2018), the p.Asp148Tyr NHLRC2 may have retained some of its physiological activity, allowing normal embryonic development both in humans and mice. *Nhlrc2*^FINCA/−^ mice retained 5% residual NHLRC2 throughout the body, including NPCs.

We compared the proteomes of *Nhlrc2*^FINCA/−^ and *Nhlrc2*^+/+^ NPCs to study the functional processes affected by NHLRC2 deficiency in developing neurons. Proteomics identified 19 proteins that were significantly affected by the *Nhlrc2*^FINCA/−^ genotype. Cellular compartment GO enrichment analysis of the altered proteins revealed a strong association with vesicles, extracellular exosomes, protein-containing complexes, and cytosol. Consistent with our findings, NHLRC2 has been connected previously to forms of endocytosis, such as phagocytosis (Haney et al. 2018; Yeung et al. 2019) and multivesicular transportation (Paakkola et al. 2018) in other cell types, and when mutated, could lead to pathological aggregation of proteins. Neurodegenerative diseases are often characterized by protein aggregates, and, not surprisingly, mutations in endo- and autolysosomal pathway genes have been associated with several neurodegenerative disorders, such as AD, PD, and lysosomal storage disease (Wang et al. 2018; Menzies et al. 2015). Neurons aim to remove the accumulated proteins through an endosomal pathway by lysosomal degradation or by releasing them into the extracellular space via multivesicular bodies and exosomes (Kalani et al. 2014). In addition to the late-onset storage diseases mentioned above, there are neurodevelopmental disorders with multisystemic involvement where autophagy impairment has been implicated, such as Vici syndrome (Hori et al. 2017) and Rett syndrome (Sbardella et al. 2017), which partly resemble FINCA disease. Collectively, the dysfunction in vesicular trafficking may create a predisposition to neurodegeneration in FINCA disease.

The proteomic approach we used resulted not only in the identification of changes in the amount of proteins in vitro, but it also provided further information about the characteristics of these proteins revealing the presence of altered isoforms (hnRNP C2, RBM8A, PEA15) as well as fragments (UBA1, FTL1). This suggests that additional regulatory mechanisms apart from transcription and translation, such as alternative splicing, PTM, or different turnover rates may play a role in FINCA disease pathology. Whereas isoforms can have different functions, PTMs regulate protein folding or activity, subcellular targeting, and interaction with ligands or other proteins (Burkle 2001), among other functions. In this study, VCP was the only spot that showed decreased intensity in *Nhlrc2*^FINCA/−^ NPCs by proteomic analysis, and it was a central node in the protein–protein interaction analysis. Although the interaction analysis may include theoretical interactions that have not been confirmed by experimental evidence, these results made VCP an interesting hit. However, several VCP antibodies failed to recognize the spot identified by MS. This may suggest we identified a change in a specific VCP form, possibly resulting from a PTM, which changes the motility of VCP in 2D electrophoresis and prevents its recognition by antibodies. VCP is known to be regulated by a large number of PTMs (Hornbeck et al. 2014). Loss of the *Nhlrc2* plant ortholog, suppressor of quenching 1, has been found to affect the electrophoretic mobility of plastid lipocalin, due to an unidentified protein modification (Malnoë et al. 2018). This raises the question whether VCP could be a target for such a protein modification. On the other hand, *Nhlrc2*^FINCA/−^ NPCs showed a striking decrease in the amount of NHLRC2, in contrast to a more modest decrease in *Nhlrc2* mRNA expression. The introduction of the Tyr residue has been proposed to disrupt hydrogen bonding which may destabilize the conformation of the mutated NHLRC2 (Biterova et al. 2018). Mutated NHLRC2 seems to be unstable in vivo and would need to be degraded. VCP is a well-known player in the ubiquitin–proteasome pathway (Ye et al. 2005), and the decrease in VCP spot intensity could result from changes in PTMs of VCP required for the degradation of the mutated NHLRC2. Mutations in *VCP* have been connected to neurodegenerative multisystemic proteinopathies in humans, such as inclusion body myopathy with Paget disease of bone and frontotemporal dementia (IBMPBFD) (Kimonis et al. 2000), amyotrophic lateral sclerosis (ALS) (Johnson et al. 2010), and Charcot-Marie-Tooth disease type 2 (CMT2) (Gonzalez et al. 2014). VCP is involved in a large number of cellular processes, but it is also involved in endo- (Ritz et al. 2011; Ramanathan and Ye 2012; Pleasure et al. 1993) and autophagic pathways (Ju and Weihl 2010). Interestingly, VCP deficiency leads to accumulation of immature autophagic vesicles (Ju et al. 2009; Tresse et al. 2010), and IBMPFD patient myoblasts accumulate large LAMP-1 and LAMP-2 positive vacuoles and LC3-II (Tresse et al. 2010). LAMP-1 positive multilamellar bodies have also been detected in electron microscopy images of immortalized FINCA patient fibroblasts (Paakkola et al. 2018).

*Anxa6*, *Ftl1*, and *Hnrnpc* showed significant but opposite changes in mRNA levels out of the 19 proteins identified by proteomics analysis. This kind of discrepancy between mRNA and protein levels has been observed in numerous studies (Abdulghani et al. 2019; Poverennaya et al. 2017) and reflects the independent but most often compensatory regulatory mechanisms of transcription and translation. For *Hnrnpc*, the lower mRNA level could also be explained by autoregulation of its own translation, which has been described for several hnRNP family members (Wollerton et al. 2004; McGlincy et al. 2010; Buratti and Baralle 2011; Rossbach et al. 2009; Müller-McNicoll et al. 2019). The increase in the hnRNP C2 protein in *Nhlrc2*^FINCA/−^ embryonic NPCs and adult mouse hippocampus suggests a perturbation in overall RNA metabolism, which in turn may contribute to the neurodegeneration seen in FINCA disease. HnRNP C1/C2 is part of the hnRNP family, which is a large RNA-binding family contributing to multiple aspects of nucleic acid metabolism. Dysregulation of RNA homeostasis has been suggested as a common feature of neurodegenerative diseases, where RNA-binding proteins (RBPs) play a crucial role (Wolozin and Ivanov 2019; Conlon and Manley 2017). RBPs often have low-complexity domains, which are thought to facilitate stress and P body formation, but also make RBPs prone to self-aggregate (March et al. 2016). VCP, in contrast, has been shown to function in the clearance of stress and P bodies (Buchan et al. 2013). hnRNPs have been recognized in several common late-onset neurodegenerative diseases, such as spinal muscular atrophy (SMA), ALS, AD, and frontotemporal dementia (Wolozin and Ivanov 2019; Geuens et al. 2016; Purice and Taylor 2018). Interestingly, an increase in hnRNP C2 was detected in the hippocampus of adult *Nhlrc2*^FINCA/−^ mice, but not in the cerebellum or brainstem. The hippocampal CA1 pyramidal cell layer was also one of the brain regions of adult mice with the highest expression of *Nhlrc2* according to our ISH data. Altogether, this data suggests that NHLRC2 may play an important role in hippocampal pyramidal cells, which were also affected by neurodegeneration in FINCA patients (Uusimaa et al. 2018).

KI models often show a milder phenotype than human patients (Dawson et al. 2018). Here, the *Nhlrc2*^FINCA/−^ mice showed normal growth compared to their wild-type litter mates and did not develop a severe disease phenotype as described in FINCA patients. Environmental factors such as pathogens or the specific genetic background may play an additional role in triggering a more severe outcome of the disease in mice (Doetschman 2009; Keane et al. 2011; Qosa and Kaddoumi 2016). Interestingly, NHLRC2 has recently been connected to immunological responses via initiation of phagocytosis in human macrophages, possibly through its effect on RhoA/Rac1 signalling, which controls actin polymerization and filopodia formation (Haney et al. 2018; Yeung et al. 2019). In addition to phagocytosis, actin cytoskeleton dynamics are important for many other cellular functions requiring remodelling of plasma membrane, or biogenesis and transport of vesicular cargo, such as cell motility, cytokinesis, endocytosis, and autophagy (Anitei and Hoflack 2012). Our proteomic analysis revealed changes in several proteins connected to actin dynamics in *Nhlrc2*^FINCA/−^ NPCs, including BRICK1 (Eden et al. 2002; Gautreau et al. 2004), costars family protein ABRACL (Pang et al. 2010), plastin-3 (Bretscher 1981), prefoldin subunit 2 (Tsao et al. 2006; Martin-Benito et al. 2002), and VCP (Nalbandian et al. 2012; Chan et al. 2012; Shah and Beverly 2015). Finally, *Nhlrc2*^FINCA/−^ mice, housed in specific pathogen-free conditions, may have lacked an immunological component important for triggering the FINCA disease onset, where recurrent infections were present in human patients.

## Conclusions

Even though mouse models of neurodegeneration seldom show the whole spectrum of the human disease, they have proven to be invaluable tools for studying the molecular pathogenesis of neurodegeneration (Dawson et al. 2018; Leung and Jia 2016). Our results suggest for the first time that the *Nhlrc2*^FINCA/−^ genotype results in dysregulation of RNA homeostasis in mouse neurons, which could ultimately contribute to the pathophysiology of neurodegeneration in FINCA disease. Further studies on human neurons are still required, but our findings presented here open future possibilities in the search for treatment and diagnosis tools for FINCA and other relevant, more common neurodegenerative diseases (Samie and Xu 2014; Spilman et al. 2010; Caccamo et al. 2014; Kim et al. 2014; Cheng et al. 2015; Lugli et al. 2015). Considering the broad spectrum of manifestations of FINCA disease, including lung and liver fibrosis, chronic hemolytic anemia, and cerebral angiomatosis, the FINCA mouse model described here offers an interesting and biologically relevant research tool for a variety of future studies.

## Supplementary information

**Additional file 1**: Detailed description of the materials and methods, supplementary figures S1- S11, and supplementary tables S1-S6. Materials and methods include generation of FINCA mouse, used animals, genotyping, Sanger equencing, histology, blood analysis, ISH, NPC culture, 2DE-DIGE, mass spectrometry, qPCR, immunoblotting, ICC, data analysis, and statistical considerations. Fig. S1 Genotyping and Sanger sequencing of three founders obtained from microinjections of Cas9 ribonucleoprotein and ssODN into mouse zygotes. Fig. S2 SDS-PAGE immunoblotting showing decrease of NHLRC2 in different brain regions and different tissues of Nhlrc2^FINCA/−^ mice compared to wild type mice. Fig. S3 SDS-PAGE immunoblotting comparing the amount of NHLRC2 between wildtype, homozygous Nhlrc2^FINCA/FINCA^ and compound heterozygous Nhlrc2^FINCA/−^ mice. Fig. S4 Representative images of Nhlrc2^+/+^ and Nhlrc2^FINCA/−^ mouse lung and liver sections. Fig. S5 NPC isolation and culture. Fig. S6 Representative 2D gel of NPCs (wild type). Fig. S7 STRING network analysis of identified proteins. Fig S8 SDS-PAGE immunoblot and 2D gel immunoblotof Nhlrc2^+/+^ and Nhlrc2^FINCA/−^ NPC lysates with VCP antibodies. Fig. S9 hnRNP C2 ICC image of Nhlrc2^+/+^ and Nhlrc2^FINCA/−^ NPCs showing normal cellular localization. Fig. S10 SDS-PAGE immunoblotting of Nhlrc2^+/+^ and Nhlrc2^FINCA/−^ cerebellum and brainstem. Fig. S11 ISH of Nhlrc2^FINCA/−^ mouse brain. Table S1 Genotyping primers. Table S2 qPCR primers. Table S3 Genotype distribution of Nhlrc2^+/+^ and Nhlrc2^FINCA/−^ mouse offspring. Table S4 Blood values of Nhlrc2^+/+^ and Nhlrc2^FINCA/−^ mice. Table S5 Detailed statistical and MS data about the proteins identified from 2DE-DIGE. Table S6. qPCR results of expression levels of genes identified in 2DE-DIGE.

**Additional file 2**: Unedited full images of the immunoblots for Fig. 2, Fig. 4, Fig. S2, Fig. S3, Fig S8, and Fig S10.

## Data Availability

Majority of the data generated or analysed during this study are included in this published article [and its additional information files]. Further information and additional datasets are available from the corresponding author on reasonable request.

## Abbreviations

NHLRC2: NHL repeat-containing 2
KI: Knockin
PD: Parkinson’s disease
AD: Alzheimer’s disease
Trx: Thioredoxin
KO: Knockout
ROS: Reactive oxygen species
NPC: Neuronal precursor cell
qPCR: Quantitative polymerase chain reaction
2D-DIGE: Two-dimensional difference gel electrophoresis
MS: Mass spectrometry
VCP: Transitional endoplasmic reticulum ATPase
FDR: False discovery rate
ANXA6: Annexin A6
FTL1: Ferritin light chain 1
hnRNP: Heterogeneous nuclear ribonucleoprotein
PTM: Post-translational modification
ICC: Immunocytochemistry
ISH: In situ hybridization
MAG: Myelin associated glycoprotein
MBP: Myelin basic protein
IBMPBFD: Inclusion body myopathy with Paget disease of bone and frontotemporal dementia
ALS: Amyotrophic lateral sclerosis
CMT2: Charcot-Marie-Tooth disease type 2
RBP: RNA-binding proteins
SMA: Spinal muscular atrophy
