# Supplementary material for: Variant in NHLRC2 leads to increased hnRNP C2 in developing neurons and the hippocampus of a mouse model of FINCA disease
Source: Mol Med. 2020 Dec 9;26:123. doi: 10.1186/s10020-020-00245-4 (PMC7724728; doi:10.1186/s10020-020-00245-4)
Supplement: Supplementary file 1 — Additional file 1: Detailed description of the materials and methods, supplementary figures S1- S11, and supplementary tables S1-S6. Materials and methods include generation of FINCA mouse, used animals, genotyping, Sanger equencing, histology, blood analysis, ISH, NPC culture, 2DE-DIGE, mass spectrometry, qPCR, immunoblotting, ICC, data analysis, and statistical considerations. Fig. S1 Genotyping and Sanger sequencing of three founders obtained from microinjections of Cas9 ribonucleoprotein and ssODN into mouse zygotes. Fig. S2 SDS-PAGE immunoblotting showing decrease of NHLRC2 in different brain regions and different tissues of Nhlrc2FINCA/− mice compared to wild type mice. Fig. S3 SDS-PAGE immunoblotting comparing the amount of NHLRC2 between wildtype, homozygous Nhlrc2FINCA/FINCA and compound heterozygous Nhlrc2FINCA/− mice. Fig. S4 Representative images of Nhlrc2+/+ and Nhlrc2FINCA/− mouse lung and liver sections. Fig. S5 NPC isolation and culture. Fig. S6 Representative 2D gel of NPCs (wild type). Fig. S7 STRING network analysis of identified proteins. Fig S8 SDS-PAGE immunoblot and 2D gel immunoblotof Nhlrc2+/+ and Nhlrc2FINCA/− NPC lysates with VCP antibodies. Fig. S9 hnRNP C2 ICC image of Nhlrc2+/+ and Nhlrc2FINCA/− NPCs showing normal cellular localization. Fig. S10 SDS-PAGE immunoblotting of Nhlrc2+/+ and Nhlrc2FINCA/− cerebellum and brainstem. Fig. S11 ISH of Nhlrc2FINCA/− mouse brain. Table S1 Genotyping primers. Table S2 qPCR primers. Table S3 Genotype distribution of Nhlrc2+/+ and Nhlrc2FINCA/− mouse offspring. Table S4 Blood values of Nhlrc2+/+ and Nhlrc2FINCA/− mice. Table S5 Detailed statistical and MS data about the proteins identified from 2DE-DIGE. Table S6. qPCR results of expression levels of genes identified in 2DE-DIGE. [file 10020_2020_245_MOESM1_ESM.pdf]

Variant in NHLRC2 leads to increased hnRNP C2 in developing neurons and the hippocampus of a mouse model of FINCA disease

Anniina E. Hiltunen [1, 2], Salla M. Kangas [1, 2], Steffen Ohlmeier [3], Ilkka Pietilä [1,4], Jori Hiltunen [1], Heikki Tanila [5], Colin McKerlie [6, 7], Subashika Govindan [8], Hannu Tuominen [9, 10], Riitta Kaarteenaho [11, 12], Mikko Hallman [1], Johanna Uusimaa [1, 13], Reetta Hinttala [1, 2].

[1] Medical Research Center Oulu and PEDEGO research unit, University of Oulu and Oulu University Hospital, Oulu, Finland

[2] Biocenter Oulu, University of Oulu, Oulu, Finland

[3] Proteomics Core Facility, Biocenter Oulu, Faculty of Biochemistry and Molecular Medicine, University of Oulu, Oulu, Finland

[4] Department of Immunology, Genetics and Pathology, Science for Life Laboratory, Uppsala University, Rudbeck Laboratory, Uppsala, Sweden

[5] A.I. Virtanen Institute, University of Eastern Finland, Kuopio, Finland

[6] The Hospital for Sick Children, Toronto, Canada

[7] Faculty of Medicine, University of Toronto, Toronto, Canada

[8] Tissue Engineering Laboratory, Hepia/HES-SO, University of Applied Sciences Western Switzerland, Geneva, Switzerland

[9] Department of Pathology, Cancer and Translational Medicine Research Unit, University of Oulu, Oulu, Finland

[10] Department of Pathology, Oulu University Hospital, Oulu, Finland

[11] Research Unit of Internal Medicine, Respiratory Research, University of Oulu, Oulu, Finland

[12] Medical Research Center Oulu and Unit of Internal Medicine and Respiratory Medicine, Oulu University Hospital, Oulu, Finland

[13] Clinic for Children and Adolescents, Paediatric Neurology Unit, Oulu University Hospital, Oulu, Finland

Corresponding author:
Anniina E. Hiltunen, M.Sc.
PO Box 5000
FIN-90014 University of Oulu

Email: anniina.hiltunen@oulu.fi

SUPPLEMENTAL MATERIAL

MATERIAL AND METHODS

Animals

Heterozygous FINCA mice (C57BL/6N-*Nhlrc2*^{em1Rthl}), hereafter referred to as *Nhlrc2*$^{FINCA/+}$ mice, harbouring the FINCA patient variant (c.442G>T, p.Asp148Tyr) were generated by clustered regularly interspaced short palindromic repeat/CRISPR-associated 9 (CRISPR/Cas9) based genome editing [1–4]. The CRISPR RNA (crRNA) used (available upon request) was designed with the CRISPR finder tool at the Wellcome Trust Sanger Institute Genome Editing webpage (sanger.ac.uk/htgt/wge; WGE CRISPR ID: 427801623). Single-stranded 158 nt oligodeoxynucleotide (ssODN) (IDT, Coralville, IA, USA; available upon request) containing the patient mutation (NM_025811: c.442G>T) and a silent mutation (NM_025811: c.408C>A), removing the protospacer adjacent motif (PAM) and adding a new TatI restriction site, was used as a homology-directed repair -template. Preassembled crRNA/trans-activating crRNA (tracrRNA) (Sigma-Aldrich, St. Louis, MO, USA)/Cas9 (IDT, Coralville, IA, USA) ribonucleoprotein complex, together with the ssODN, was injected into C57BL/6NCrl mouse zygotes. Microinjection, sperm cryopreservation [5], and *in vitro* fertilization (IVF) (https://www.infrafrontier.eu/knowledgebase/protocols/cryopreservation-protocols, 24.3.20) were performed at the Biocenter Oulu Transgenic Core Facility, University of Oulu, Finland. Embryos were transferred to pseudo-pregnant CD1 females, with an appropriate number of wild-type embryos to ensure pregnancy. Pups were screened by Sanger sequencing and TatI digestion of a PCR product from the modified region. Founders, carrying mutations, were mated with wild-type C57BL/6NCrl mice, and F1 pups were analyzed in the same manner. *Nhlrc2*$^{FINCA/+}$ mice were crossed with heterozygous *Nhlrc2* KO mice (C57BL/6N-A^{tm1Brd}*Nhlrc2*$^{tm1a(KOMP)Wtsi}$/WtsiOulu, EMMA ID: EM:10219) [6], referred to as *Nhlrc2*$^{-/+}$, to obtain compound heterozygous *Nhlrc2*$^{FINCA/-}$ mice and wild-type *Nhlrc2*$^{+/+}$ litter mates.

Animals were bred in a specific pathogen free (SPF) facility, and all experiments were carried out in the conventional unit of the Oulu Laboratory Animal Centre. Apart from mouse norovirus in the conventional unit, the facility was free of all primary mouse pathogens listed in the Federation of European Laboratory Animal Science Associations (FELASA) recommendations [7]. The animal holding temperature was 21 ± 2°C with 55% (40-70%) humidity in the SPF facility and 40% humidity in the conventional unit. The light/dark cycle was 12 h light (06:00−18:00) and 12 h dark (18:00−06:00). Chow in the conventional unit was Teklad Global Rodent diet T.2018C.12 (Harlan Teklad, USA) and irradiated Teklad Global Rodent diet T.2918 (Harlan Teklad, USA) in the SPF unit. Mice were housed on Aspen chips and shavings (Tapvei, Harjumaa, Estonia), and a nest box (Mouse-house II) and cube tunnel were provided in every cage for enrichment. Adult animals were euthanized using carbon dioxide sedation and cervical dislocation or by cervical dislocation under terminal anesthesia (1 mg/kg medetomidine (Orion, Espoo, Finland) and 75 mg/kg ketamine (Intervet, Espoo, Finland)), sub cutaneous injection) after blood sample collection. Embryos were euthanized by decapitation. DNA was isolated from earmark biopsies taken at weaning or from tail clips of embryos.

All animal experiments were approved by the Regional State Administrative Agency of Southern Finland (ESAVI/5236/04.10.07/2016, ESAVI/33827/2019). The animal care and experimental procedures were conducted according to the national legislation and the EU Directive 2010/63/EU.

Genotyping and Sanger sequencing

All primers used for genotyping by polymerase chain reaction (PCR) or Sanger sequencing are listed in Table S1 of the supplementary material. PCR was performed using a Phire Hot Start II Polymerase (Thermo Fisher Scientific, Waltham, MA, USA) and a Piko Thermal Cycler (Thermo Fisher Scientific, Vantaa, Finland). The PCR products were sequenced using an ABI3500xL Genetic Analyzer at the Biocenter Oulu Sequencing Center and analyzed using Sequencher 5.0 software (Gene Codes Corporation Ann Arbor, MI, USA). The PCR products were run on a 1.5% agarose gel (BioNordika, Helsinki, Finland) and detected with SYBR safe DNA gel stain (Invitrogen, Carlsbad, CA, USA).

The CRISPR/Cas9-edited mice were genotyped using Crispr_F (4) and Crispr_R (5) primers. For founders and first-generation (F1) pups, the PCR product was sequenced and digested with TatI (Thermo Fisher Scientific, Vilnius, Lithuania) restriction enzyme to determine the insertion of the ssODN. Subsequent generations were genotyped by TatI digestion alone.

The presence of the *Nhlrc2* wild-type allele and the *Nhlrc2* KO first allele were determined with PCR using wild-type *Nhlrc2*-specific primers (Nhlrc2_119354_F and Nhlrc2_119354_R) and primers for the LacZ cassette included in the KO first allele.

Histology and blood analysis

Nhlrc2$^{FINCA/-}$ mice (6 males and 6 females) and *Nhlrc2*$^{+/+}$ mice (6 males and 5 females) were weighed weekly until 8 weeks of age and then monthly or every other month until 32 weeks of age. Blood samples were collected from the retro-orbital venous plexus under terminal anesthesia into EDTA tubes (Sartsted, Sarsted, Gemany) with EDTA disodium dihydrate (Merck, Netherlands) coated hematocrit capillary tubes (Heinz Herenz medizinalbedarf, Hamburg, Germany). Hemoglobin was measured using a Hemocue Hb 201+ (HemoCue AB, Ängelholm, Sweden). Hematocrit was measured by centrifugation of blood samples in 75 µL microhematocrit tubes using a microhematocrit centrifuge (Clay-Adams, New York, NY, USA) and measured with a microhematocrit reader (Clay-Adams, New York, NY, USA).

Fresh tissues were collected immediately after euthanasia and fixed by immersion in 10% neutral buffered formalin (FF-Chemicals, Haukipudas, Finland). The intact brain was fixed for 48h, and the middle region of the left lung lobe and a portion of the median lobe of the liver were fixed for 24 h, at room temperature under agitation. After fixation, the tissues were washed three times in Dulbecco's phosphate-buffered saline (PBS; Corning, Manassas, VA, USA) and transferred to 70% ethanol. Brains were weighed after fixation. Tissues were processed using Tissue Tek VIP 5 Jr, embedded into paraffin, and sectioned at 5 µm (Microm, Walldorf, Germany). Brain sections were stained with hematoxylin and eosin (HE) and Luxol Fast Blue [8]. Lung sections were stained with H&E or modified Masson's trichrome staining to highlight collagen content. Whole slide images were acquired with a NanoZoom S60 scanner (Hamamatsu, Hamamatsu city, Japan) at 20x magnification and NDP.view2 (Hamamatsu, Hamamatsu city, Japan) was used for image analysis.

In situ hybridization (ISH)

An RNAscope 2.5 HD RED Assay (ACD, Newark, CA, USA) was used according to the manufacturer's instructions for detection of *Nhlrc2* mRNA from 5 µm paraffin-embedded brain tissue sections [9]. Target retrieval was performed by boiling the sections at 98°C for 15 minutes in RNAscope target retrieval reagent using a KOS Microwave HistoSTATION (Milestone, Sorisole, Italy). Positive and negative control probes, mm-Ppib and mm-dapB, respectively, were used to optimize the protocol. The *Nhlrc2* probe used was mm-Nhlrc2 (591971, ADC). Hematoxylin (Sigma-Aldrich, MO, USA)

was used to stain the nuclei, and coverslips were mounted with EcoMount (Biocare Medical, CA, USA). Whole slide images were acquired with a NanoZoom S60 scanner (Hamamatsu, Hamamatsu city, Japan) at 40x magnification, and NDP.view2 (Hamamatsu, Hamamatsu city, Japan) was used for image analysis.

Neuronal precursor cell (NPC) culture

Heterozygous *Nhlrc2*$^{FINCA/+}$ and *Nhlrc2*$^{+/-}$ mice were mated, and the presence of a vaginal plug was regarded as E 0.5. At E13.5, the embryos were decapitated, and the cortices were dissected into 250 µL of ice-cold Hanks' Balanced Salt solution without calcium or magnesium (Gibco, Paisley, UK). The tissue was trypsinized by adding 50 µL of 0.05% trypsin (Lonza, Vervier, Belgium) and incubating for 20 minutes at 37°C. The trypsin was inactivated by adding 200 µL of fetal bovine serum (Pan-Biotech, Aidenbach, Germany). After 5 minutes of centrifugation at 200 *g*, to remove the supernatant, the cells were triturated into pre-warmed neurobasal medium supplemented with 2% B27 (Thermo Fisher Scientific, Grand Island, NY, USA), 10 u/mL penicillin/streptomyosin (Sigma-Aldrich, St. Louis, MO, USA), 0.5 mM sodium pyruvate (Gibco, Paisley, UK), and 1mM L-alanyl-L-glutamine (Corning, Manassas, VA, USA). The cells were filtered through a 35 µm nylon mesh (Corning Science Mexico, Tamaulipas, Mexico) and 0.5×10^6 cells were plated onto 12-well plates coated with laminin (Sigma-Aldrich, St. Louis, MO, USA) and poly-l-lysine (Sigma-Aldrich, St. Louis, MO, USA). The cells were kept in a cell culture incubator (37°C, 5% CO_2; BINDER, Tuttlingen, Germany). The medium was changed on the second and fifth days of culture. The NPCs were harvested after 6 days *in vitro* (DIV) by trypsinzation. The cell pellets were stored at −70°C until analysis.

Two-dimensional difference gel electrophoresis (2D-DIGE)

Nhlrc2$^{FINCA/-}$ (n=6) and *Nhlrc2*$^{+/+}$ (n=6) mouse neuronal precursor cell pellets stored at −70°C were purified by acetone precipitation, and proteins were solubilized in urea buffer (7 M urea, 2 M thiourea, 4 % [w/v] CHAPS, 30 mM Tris, pH 8.0). The amount of protein was determined with a Bradford-based assay according to the manufacturer's instructions (Roti®-Nanoquant, Carl Roth, Karlsruhe, Germany), and aliquots were stored at −20°C. Protein labelling was performed with the "saturation DIGE" kit (GE Healthcare, Piscataway, NJ, USA) according to the manufacturer's protocol with a sample-specific adaptation of the dye ratio (5 µg protein/0.25 nmol dye). Immobilized pH gradient strips (pH 4−7, 24 cm; GE Healthcare) were incubated overnight in 650 µL rehydration buffer (7 M urea, 2 M thiourea, 4% [w/v] CHAPS, 20 mM [w/v] DTT, 0.5% [v/v] carrier ampholytes 3−10, Complete Mini protease inhibitor cocktail). After anodic sample cup-loading, the proteins were separated by isoelectric focusing in the IPGphor 3 system (GE Healthcare) under paraffin oil for 80 kVh. SDS-PAGE was performed overnight in polyacrylamide gels (12.5%) with the Ettan DALT II system (GE Healthcare) at 1−2 W per gel and 12°C. Fluorescence signals were detected with a Typhoon 9400 (GE Healthcare). 2-D gels were analyzed with Delta2D 4.6 (Decodon, Greifswald, Germany).

Mass spectrometry (MS)

For protein identification, additional 2D gels were run with higher amounts of unlabeled protein (400−800 µg) combined with 5 µg Cy3-labeled internal standard. After detection of the fluorescence signals and silver staining, labeled and unlabeled protein patterns were matched with the 2-DE image analysis software Melanie 3.09 (GeneBio, Geneva, Switzerland). Spots with correctly matched centers were excised, digested with trypsin (recombinant; Roche), and prepared as described previously [10]. The extracted and dried peptides were dissolved in 5 µL of α-Cyano-3-hydroxycinnamic acid (98%, recrystallized from ethanol-water, 5 mg/mL in 50% [v/v] acetonitrile, and 0.1% [v/v] TFA), and 0.5 µL was applied onto the sample plate using the dried-droplet method. Peptide masses were measured with an UltrafleXtreme MALDI-TOF/TOF (Bruker, Billerica, MA, USA). Proteins were identified according to their spot-

specific peptide mass fingerprints and peptide sequences with the bioinformatic tool BioTools Version 3.2 (Bruker; search parameters: MS tolerance: 100 ppm, MSMS tolerance: 0.7 Da, enzyme: trypsin, engine: Mascot version 2.4.0, database: NCBInr, modifications: carbamidomethyl (Cys) and optional oxidation of Met, up to 1 missed cleavage).

Quantitative PCR (qPCR)

NPC samples from three *Nhlrc2*$^{FINCA/-}$ and three *Nhlrc2*$^{+/+}$ embryos were collected for qPCR analysis. RNA isolation was carried out using an RNeasy Plus Mini Kit (Qiagen, Hilden, Germany), and a QuantiTect Reverse Transcription Kit (Qiagen, Hilden, Germany) was used for complementary cDNA synthesis. qPCR primers (Table S2) were designed using NCBI Primer-BLAST [11], and the T_m used was 60°C. RPL13A [12,13] and PKG1 [14,15] were used as reference genes. qPCR was performed according to the manufacturer's instructions (IQ™ SYBR Green Supermix, Bio-Rad, Hercules, CA, USA) with a CFX Connect™ Real-Time System (Bio-Rad, Hercules, CA, USA).

Immunoblotting

Tissues were transferred to liquid nitrogen immediately after dissection and homogenized for immunoblotting as described previously [16]. NPCs were solubilized after trypsinization for 40 minutes on ice in 1.5% Dodecyl β-d-maltopyranoside (Sigma-Aldrich, St. Louis, MO, USA) with protease inhibitor cocktail (Thermo Fisher Scientific, Rockford, IL, USA). The lysate was centrifuged for 20 minutes at 20,000 *g* and +4°C, after which the supernatant containing the proteins was transferred to a new microcentrifuge tube. The amount of protein in cell and tissue lysates was measured using a Coomassie protein assay (Thermo Fisher Scientific, Rockford, IL, USA) and FLUOstar Omega microplate reader (BMG LabTech, Ortenberg, Germany).

Fifteen micrograms of protein per NPC sample, or 20µg per tissue sample, and a prestained protein ladder of 10−180 kDa (Thermo Fisher Scientific, Vilnius, Lithuania), were loaded onto a 4–20% polyacrylamide gel (Bio-Rad, Hercules, CA, USA). Electrophoresis was run at 100V for 1.5 h, or for hippocampus samples for 3 h, in Tris/Glycine/SDS buffer (Bio-Rad, Hercules, CA, USA). Proteins were transferred onto a nitrocellulose membrane (Bio-Rad, Hercules, CA, USA) using a Trans-blot Turbo transfer system (Bio-Rad, Singapore) and Ponceaus S (Biotium, Fremont, CA, USA) was used to verify even transfer of proteins. Five percent non-fat dry milk (Valio, Finland) in Tris-buffered saline (Medicago, Uppsala, Sweden) with 0.01% tween 20 (Fisher Scientific, Geel, Belgium) was used to block unspecific binding. Primary antibody incubations were carried out overnight at +4°C under agitation. Secondary antibodies were incubated at RT for 1 h under agitation. Chemiluminescence detection was carried out using an ECL kit (Advansta, Menlo Park, CA, USA) and an LAS-3000 Luminescent Image Analyser (Fuji Photo Film, Tokyo, Japan). Fiji software [17] was used to determine the band intensities.

Primary antibodies used were NHLRC2 antibody (Novus biologicals, NBP1-85019, 1:500), GAPDH antibody (GeneTex, GTX100118, 1:1000), VCP antibodies (Thermo Fisher Scientific, MA3-004 1:2000, Thermo Fisher Scientific PA5-29638 1:1000, Santa Cruz SC-57492 1:200), hnRNP C2 antibody (Abcam, ab 133607, 1:20,000), MAG antibody (Millipore, MAB1567, 1:500) and MBP antibody (CST, #78896, 1:1000).

Secondary antibodies used were Goat Anti-Rabbit IgG H&L horseradish peroxidase (Abcam ab97080, 1:10,000) and Goat Anti-Mouse IgG H&L horseradish peroxidase (Abcam ab6789, 1:10,000).

Immunocytochemistry (ICC)

Approximately 1.25 × 10^5 NPCs were plated on coverslips coated with laminin and poly-l-lysine and fixed 48 h later. ICC was performed as described previously [18].

Primary antibodies used were hnRNP C2 (Abcam, ab 133607, 1:500) and β3-Tubulin (D71G9) (Cell Signalling Technology, #5568, 1:200).

Secondary antibodies used were Alexa Fluor546 goat anti-RABBIT IgG (Invitrogen, A11010, 1:2000), Alexa Fluor594 goat anti-MOUSE igG (Invitrogen, A11005,1:2000). Nuclei were stained using Hoechst (Invitrogen, H3570, 1:10,000), and Phalloidin:FITC (ECM Biosciences, P40-37500, 1:500) was used for actin visualization.

Images were taken with a Leica SP8 FALCON confocal microscope (Leica Microsystems, Wetzlar, Germany) using an HC PL APO 63x/1.40 OIL CS2 DIC (oil) objective and LAS X software.

Data analysis

Protein−protein interaction analysis was performed using a STRING resource (string-db.org) [19]. The Gene Ontology (GO) resource was used for GO term enrichment analysis (geneontology.org) (Ashburner et al., 2000; The Gene Ontology Consortium, 2019). The GO analysis type used was the PANTHER (protein analysis through evolutionary relationships) Overrepresentation Test (Released 20200407) with annotation version GO Ontology database Released 2020-03-23, test type FISHER, and the false discovery rate (FDR) correction. The reference list used was *Homo sapiens* (all genes in database).

Statistical considerations

Statistical analyses were performed using Graph Pad Prism 8.0 program. Student's unpaired t-test was used for two-group comparisons, and the analysis of variance (ANOVA) was used for repeated measures. Significance was set at $p < 0.05$.

REFERENCES

[1] Cong L, Ran FA, Cox D, Lin S, Barretto R, Habib N, et al. Multiplex genome engineering using CRISPR/Cas systems. Science 2013;339:819–23. https://doi.org/10.1126/science.1231143.

[2] Mali P, Yang L, Esvelt KM, Aach J, Guell M, DiCarlo JE, et al. RNA-guided human genome engineering via Cas9. Science 2013;339:823–6. https://doi.org/10.1126/science.1232033.

[3] Inui M, Miyado M, Igarashi M, Tamano M, Kubo A, Yamashita S, et al. Rapid generation of mouse models with defined point mutations by the CRISPR/Cas9 system. Sci Rep 2014;4:5396. https://doi.org/10.1038/srep05396.

[4] Jinek M, Chylinski K, Fonfara I, Hauer M, Doudna JA, Charpentier E. A programmable dual-RNA-guided DNA endonuclease in adaptive bacterial immunity. Science (80-) 2012;337:816–21. https://doi.org/10.1126/science.1225829.

[5] Ostermeier GC, Wiles M V., Farley JS, Taft RA. Conserving, Distributing and Managing Genetically Modified Mouse Lines by Sperm Cryopreservation. PLoS One 2008;3:e2792.

https://doi.org/10.1371/journal.pone.0002792.

[6] Skarnes WC, Rosen B, West AP, Koutsourakis M, Bushell W, Iyer V, et al. A conditional knockout resource for the genome-wide study of mouse gene function. Nature 2011;474:337–42. https://doi.org/10.1038/nature10163.

[7] Nicklas W, Baneux P, Boot R, Decelle T, Deeny AA, Fumanelli M, et al. Recommendations for the health monitoring of rodent and rabbit colonies in breeding and experimental units. Lab Anim 2002;36:20–42. https://doi.org/10.1258/0023677021911740.

[8] Bancroft JD ed., Stevens A. Theory and practice of histological techniques. 1990.

[9] Wang F, Flanagan J, Su N, Wang LC, Bui S, Nielson A, et al. RNAscope: A novel in situ RNA analysis platform for formalin-fixed, paraffin-embedded tissues. J Mol Diagnostics 2012;14:22–9. https://doi.org/10.1016/j.jmoldx.2011.08.002.

[10] Ohlmeier S, Mazur W, Salmenkivi K, Myllärniemi M, Bergmann U, Kinnula VL. Proteomic studies on receptor for advanced glycation end product variants in idiopathic pulmonary fibrosis and chronic obstructive pulmonary disease. Proteomics - Clin Appl 2010;4:97–105. https://doi.org/10.1002/prca.200900128.

[11] Ye J, Coulouris G, Zaretskaya I, Cutcutache I, Rozen S, Madden TL. Primer-BLAST: a tool to design target-specific primers for polymerase chain reaction. BMC Bioinformatics 2012;13:134. https://doi.org/10.1186/1471-2105-13-134.

[12] Rydbirk R, Folke J, Winge K, Aznar S, Pakkenberg B, Brudek T. Assessment of brain reference genes for RT-qPCR studies in neurodegenerative diseases. Sci Rep 2016;6:37116. https://doi.org/10.1038/srep37116.

[13] Yan Z, Gao J, Lv X, Yang W, Wen S, Tong H, et al. Quantitative Evaluation and Selection of Reference Genes for Quantitative RT-PCR in Mouse Acute Pancreatitis. Biomed Res Int 2016;2016:1–11. https://doi.org/10.1155/2016/8367063.

[14] Veazey KJ, Golding MC. Selection of Stable Reference Genes for Quantitative RT-PCR Comparisons of Mouse Embryonic and Extra-Embryonic Stem Cells. PLoS One 2011;6:e27592. https://doi.org/10.1371/journal.pone.0027592.

[15] Boda E, Pini A, Hoxha E, Parolisi R, Tempia F. Selection of Reference Genes for Quantitative Real-time RT-PCR Studies in Mouse Brain. J Mol Neurosci 2009;37:238–53. https://doi.org/10.1007/s12031-008-9128-9.

[16] Uusimaa J, Kaarteenaho R, Paakkola T, Tuominen H, Karjalainen MK, Nadaf J, et al. NHLRC2 variants identified in patients with fibrosis, neurodegeneration, and cerebral angiomatosis (FINCA): characterisation of a novel cerebropulmonary disease. Acta Neuropathol 2018;135:727–42. https://doi.org/10.1007/s00401-018-1817-z.

[17] Schindelin J, Arganda-Carreras I, Frise E, Kaynig V, Longair M, Pietzsch T, et al. Fiji: An open-source platform for biological-image analysis. Nat Methods 2012;9:676–82. https://doi.org/10.1038/nmeth.2019.

[18] Paakkola T, Salokas K, Miinalainen I, Lehtonen S, Manninen A, Kaakinen M, et al. Biallelic mutations in

human NHLRC2 enhance myofibroblast differentiation in FINCA disease. Hum Mol Genet 2018;27:4288–302. https://doi.org/10.1093/hmg/ddy298.

[19] Szklarczyk D, Gable AL, Lyon D, Junge A, Wyder S, Huerta-Cepas J, et al. STRING v11: protein–protein association networks with increased coverage, supporting functional discovery in genome-wide experimental datasets. Nucleic Acids Res 2019;47:D607–13. https://doi.org/10.1093/nar/gky1131.

[20] Ashburner M, Ball CA, Blake JA, Botstein D, Butler H, Cherry JM, et al. Gene Ontology: tool for the unification of biology. Nat Genet 2000;25:25–9. https://doi.org/10.1038/75556.

[21] The Gene Ontology Resource: 20 years and still GOing strong. Nucleic Acids Res 2019;47:D330–8. https://doi.org/10.1093/nar/gky1055.

[22] Ishikura S, Tsunoda T, Nakabayashi K, Doi K, Koyanagi M, Hayashi K, et al. Molecular mechanisms of transcriptional regulation by the nuclear zinc-finger protein Zfat in T cells. Biochim Biophys Acta - Gene Regul Mech 2016;1859:1398–410. https://doi.org/10.1016/j.bbagrm.2016.08.010.

SUPPLEMENTAL FIGURE

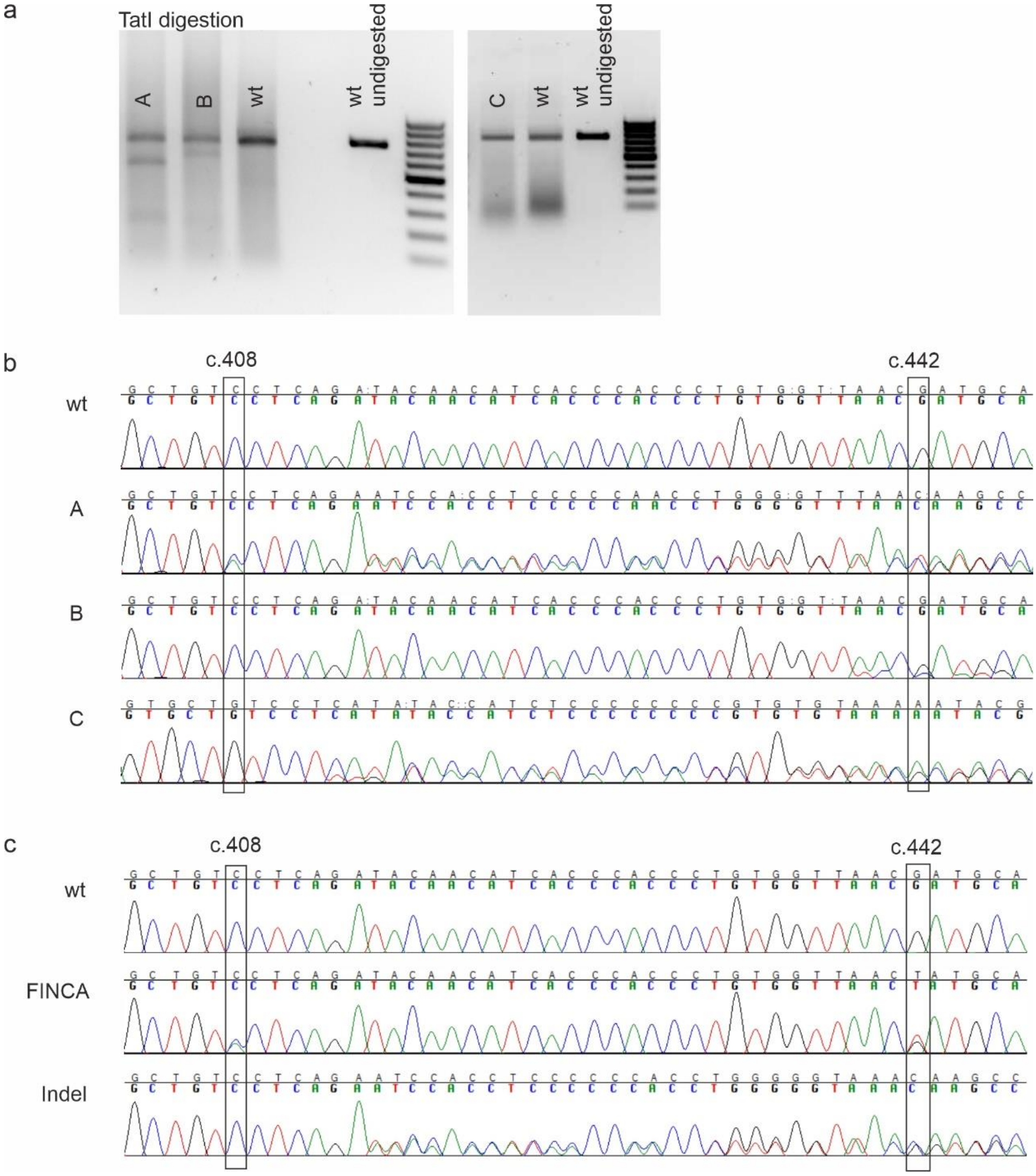

Fig. S1 FINCA allele generation. Cas9 ribonucleoprotein and the ssODN were microinjected together into C57BL/6NCrl mouse zygotes to obtain the desired modification. Of 230 zygotes injected, 21 pups were born, including three founders (A, B, C). **a** PCR product, from the edited area of founders A, B and C, was digested by TatI restriction enzyme, verifying the insertion of the ssODN. The fragments were the correct size for founder A. **b** Sanger sequencing results from the modified region of founders A, B and C. Nucleotides to be edited are highlighted (box). **c** Electropherogram images of F1 pups produced by founder A. Founder A was subfertile, and after successful IVF, this male was the only founder producing F1 pups with the correctly edited missense allele. Based on segregation of the variants, founder A was a compound heterozygote of the FINCA allele and an indel allele, both affecting the Trx-like domain of *Nhlrc2*

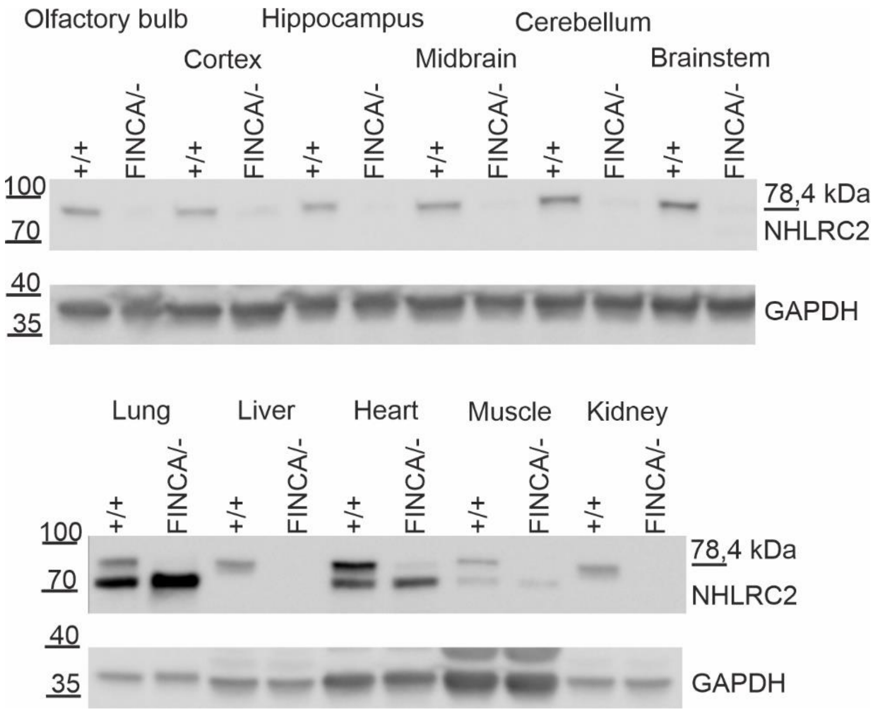

Fig. S2 SDS-PAGE immunoblotting of different brain regions; different tissues of 13-week-old *Nhlrc2*$^{FINCA/-}$ and *Nhlrc2*$^{+/+}$ female mouse show a consistent decrease in the amount of NHLRC2 protein

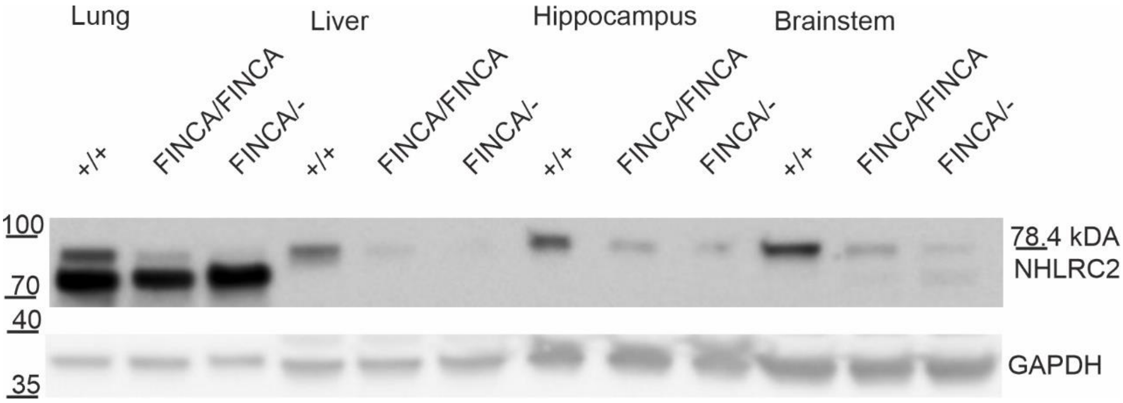

Fig. S3 SDS-PAGE immunoblotting of 13-week-old *Nhlrc2*$^{+/+}$, *Nhlrc2*$^{FINCA/FINCA}$, and *Nhlrc2*$^{FINCA/-}$ female mouse lung, liver, hippocampus, and brainstem, showing the most prominent decrease in the amount of NHLRC2 protein in *Nhlrc2*$^{FINCA/-}$ mice

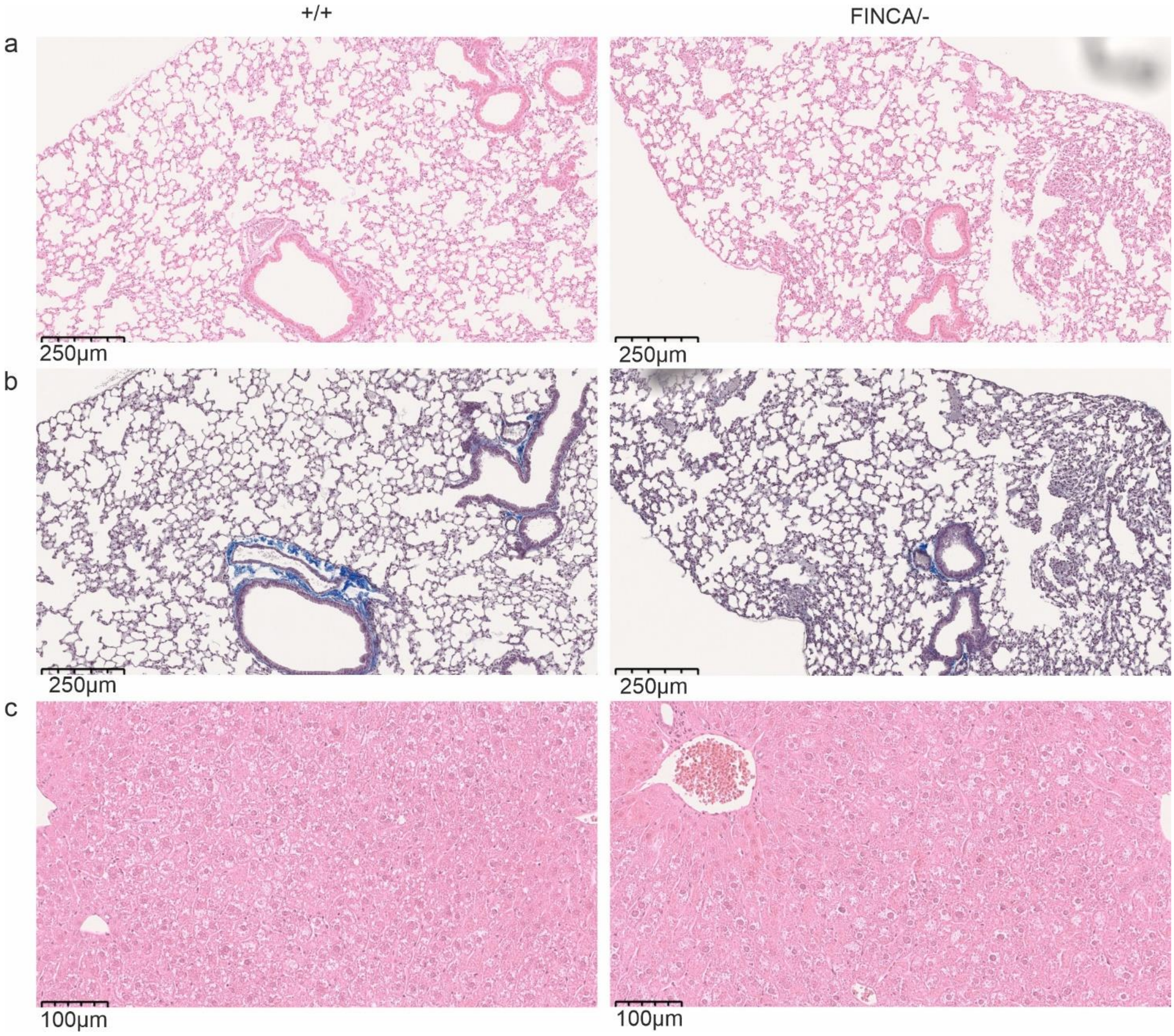

Fig. S4 Representative images of *Nhlrc2*$^{+/+}$ and *Nhlrc2*$^{FINCA/-}$ mouse lung and liver sections at 32 weeks of age. **a** Lung stained with HE. **b** Lung stained with modified Masson's trichrome showing collagen in blue. **c** Liver stained with HE

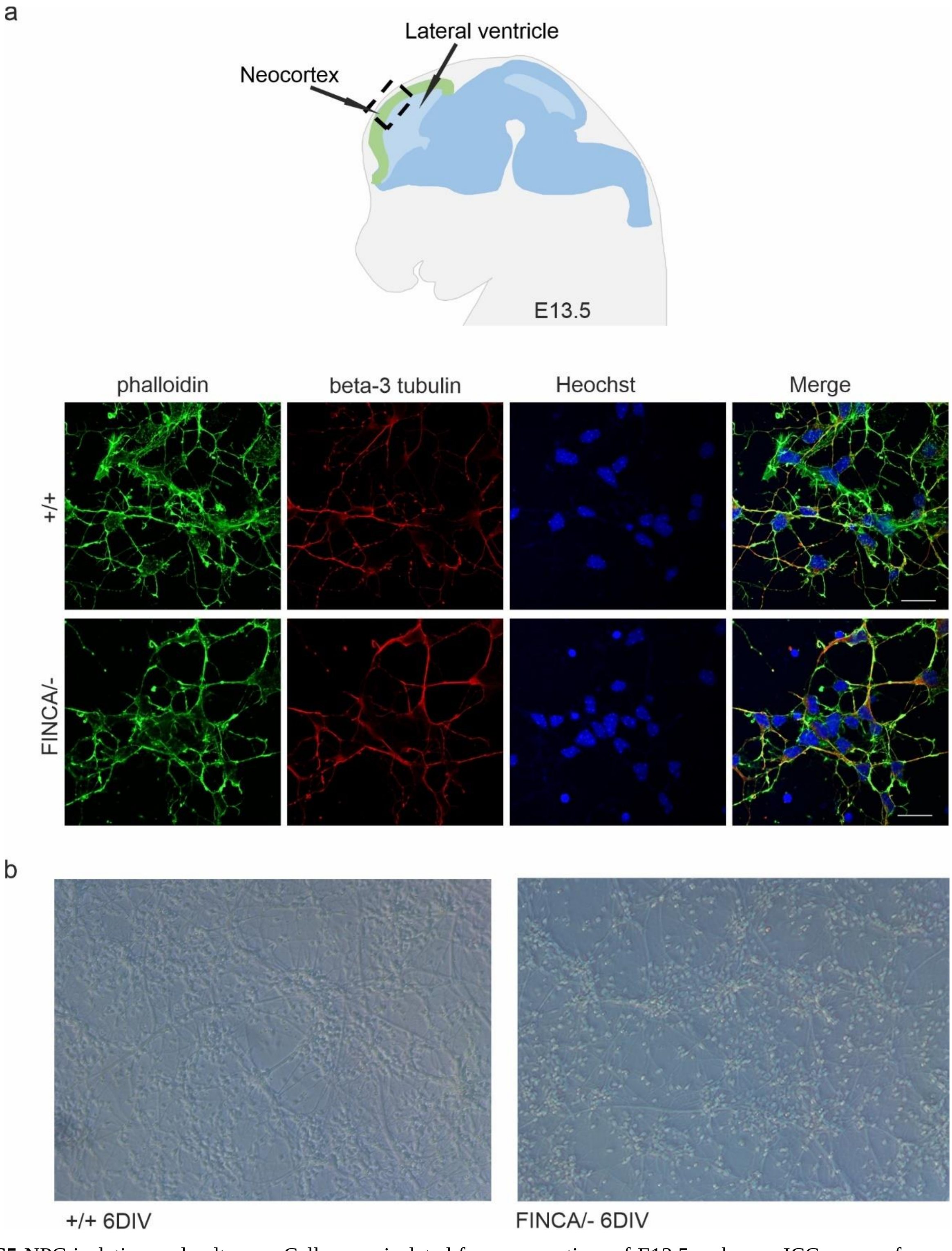

Fig. S5 NPC isolation and culture. **a** Cells were isolated from neocortices of E13.5 embryos. ICC was performed with beta3 tubulin (red), phalloidin (green) and Hoechst (blue) to verify the isolation of NPCs on cells fixed 48h after isolation. Scale bar 20µm. **b** NPCs from wild-type and mutant embryos were cultured for 6 days. *Nhlrc2*$^{FINCA/-}$ NPCs did not show any apparent phenotype

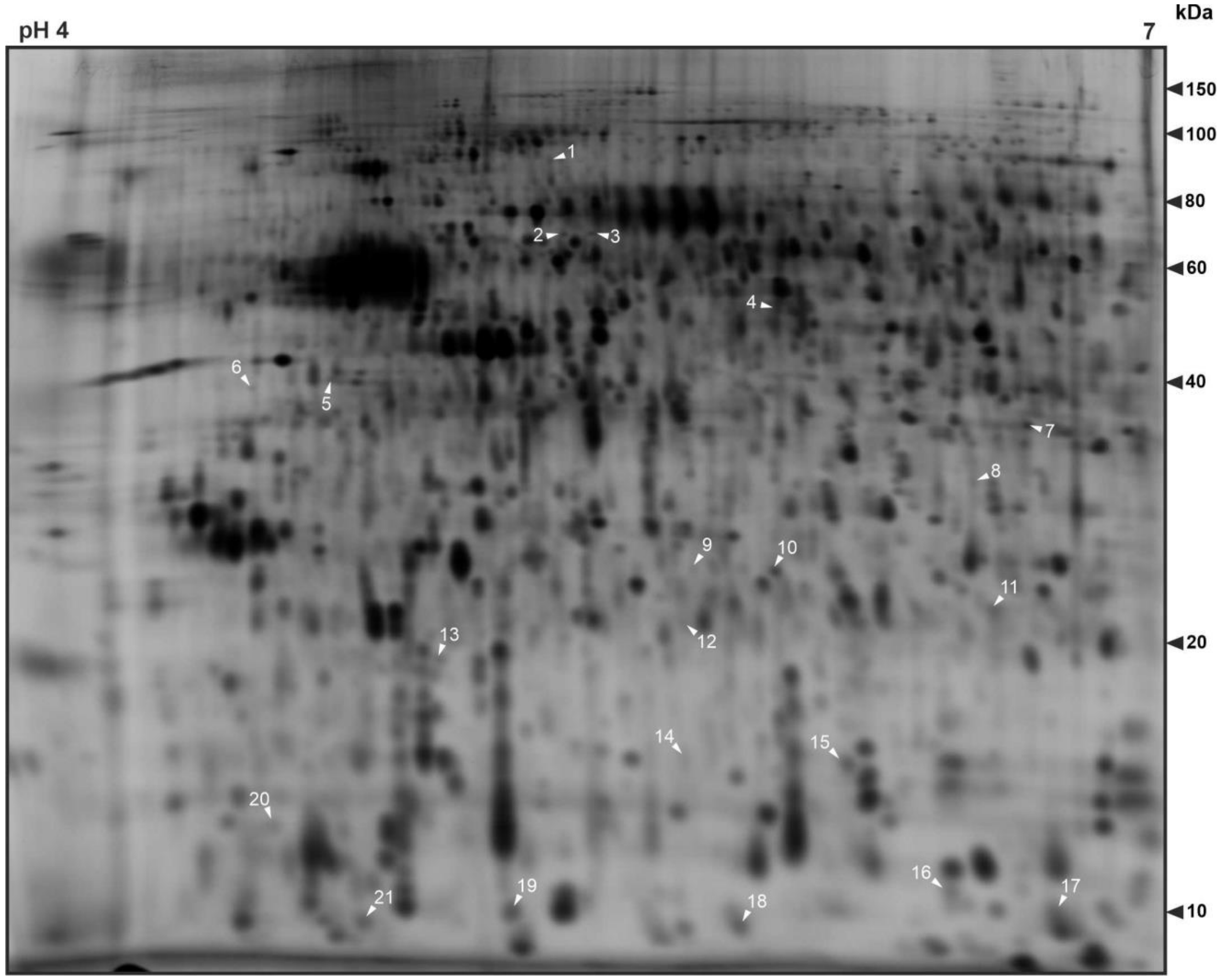

Fig. S6 Representative 2D gel of mouse neuronal precursor cells (wild-type). Proteins (5 µg) were labeled with Cys5 ("saturation DIGE"). Changed spots are indicated

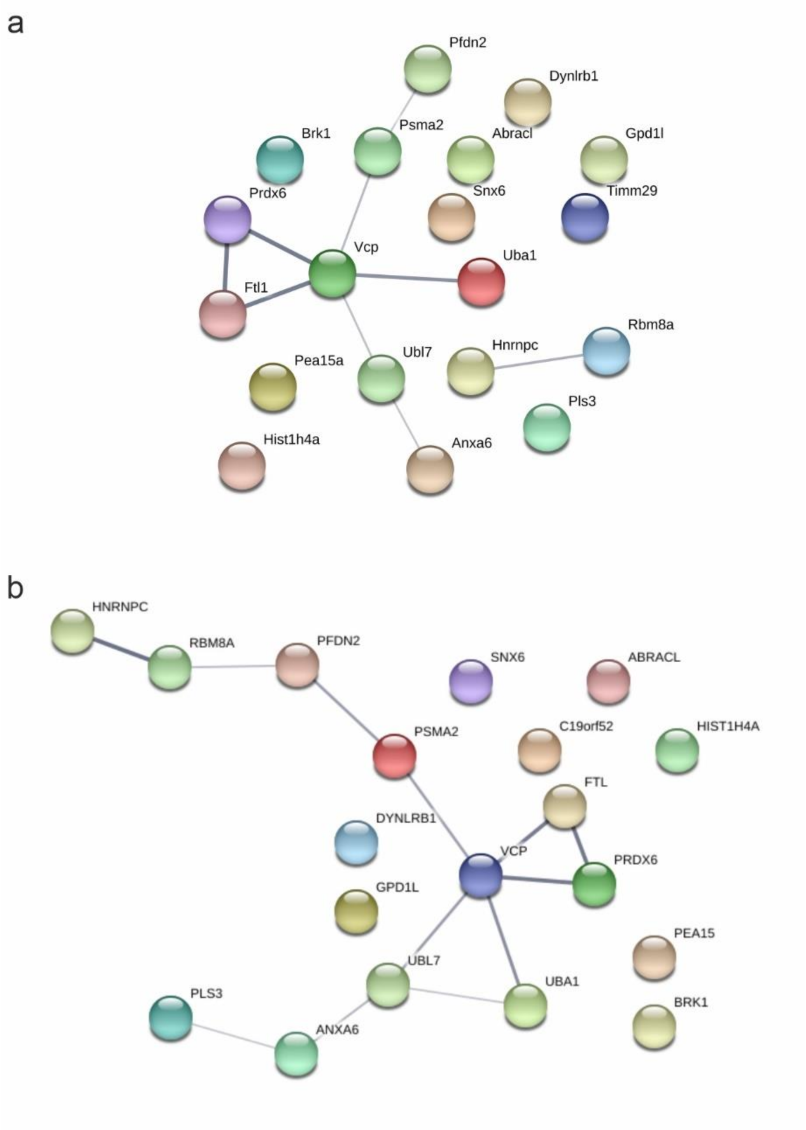

Fig. S7 STRING network analysis of identified proteins with minimum required interaction score of 0.400. **a** Mouse: PPI enrichment p-value: 0.00056. **b** Human orthologs: PPI enrichment p-value 0.00204 (https://string-db.org/, 3.12.19). Line thickness indicates the strength of the supporting data

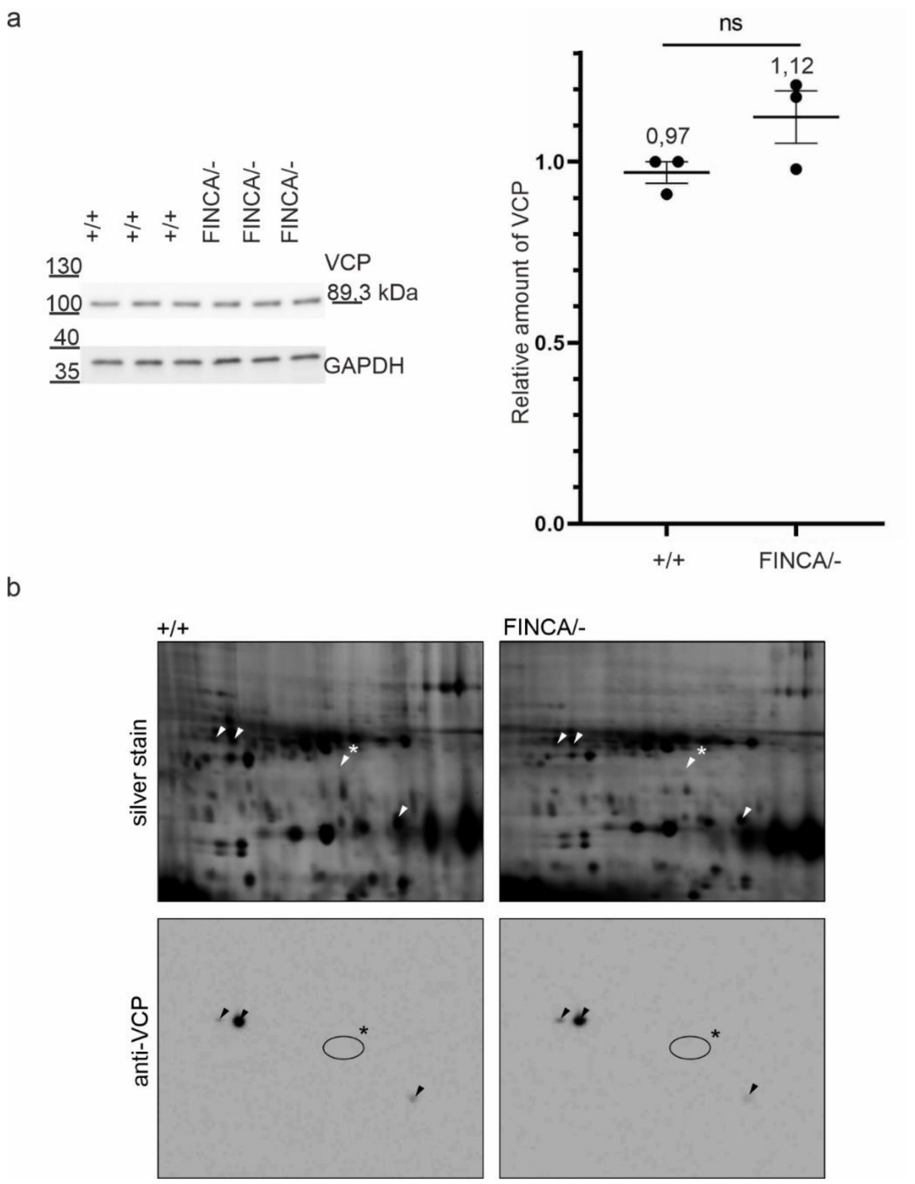

Fig. S8 Conventional SDS-PAGE and immunoblotting with 1.5% Dodecyl β-d-maltopyranoside-solubilized whole NPC extracts using three different commercially available antibodies, designed to detect amino acid sequences 290-547 or 792-806 of VCP, revealed no significant difference. To further investigate the specific spot of Vcp altered in 2D-DIGE, we performed immunoblotting directly from 2D gels. Although VCP in spot 1 was identified in independent MS analyses from six different 2D gels, which revealed a maximal sequence coverage of amino acids 2−766, the antibodies used in SDS-PAGE immunoblotting detected three other spots but not spot 1. **a** SDS-PAGE immunoblotting of VCP (PA5-29638) showed no difference between *Nhlrc2*$^{+/+}$ and *Nhlrc2*$^{FINCA/-}$ NPCs. Protein amounts are relative to one of the wild-type samples, and GAPDH was used for normalization. Individual data points, group mean, and SEM are shown. Statistical analysis was done using Student's t-test. **b** Vcp spot positions detected by 2D-DIGE and immunoblotting performed from 2D gel. Above, representative silver-stained gels of Nhlrc2$^{+/+}$ and Nhlrc2$^{FINCA/-}$ NPC samples show the position of Vcp spots detected by 2D-DIGE (*) and with the VCP antibody (PA5-29638) (arrow). Below immunoblotting performed from the gels shown above. These three spots detected by the antibodies were likewise evaluated in the 2D-DIGE gels, and all revealed a minor but not significant decrease in *Nhlrc2*$^{FINCA/-}$ in comparison to *Nhlrc2*$^{+/+}$ NPCs (data not shown)

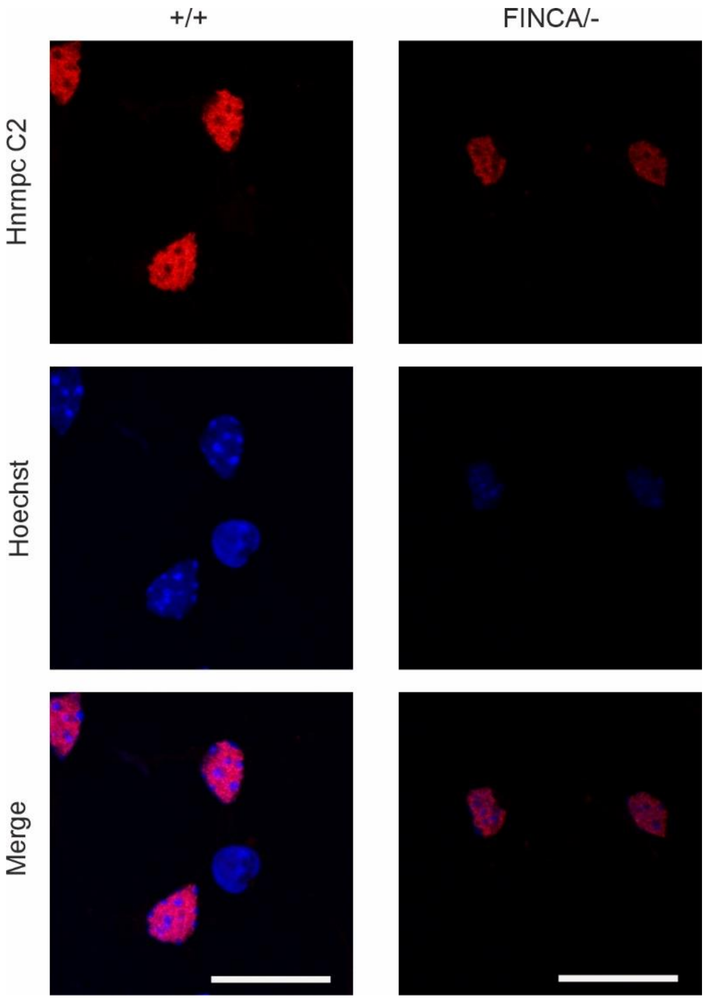

Fig. S9 hnRNP C2 has normal localization in *Nhlrc2*$^{FINCA/-}$ NPCs. NPC cells fixed 48h after isolation showing hnRNPC C2 (Red) and Hoechst (Blue). Scale bar 20 µm

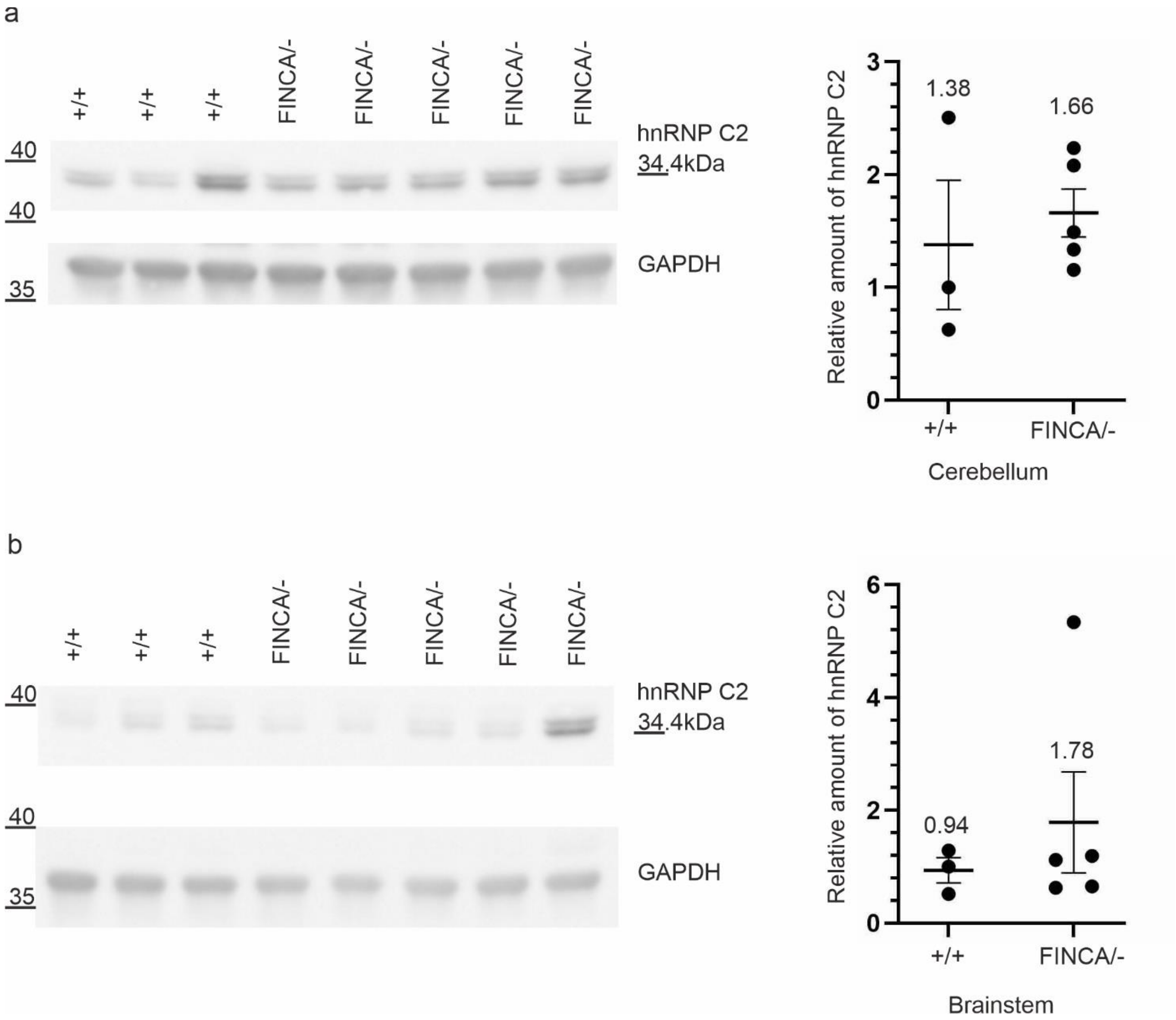

Fig. S10 Isoform C2 of Hnrnp C1/C2 immunoblotting from 13-week-old female *Nhlrc2*$^{+/+}$ (N=3) and *Nhlrc2*$^{FINCA/-}$ (N=5) mouse. **a** cerebellum, and **b** brainstem lysates. There were no significant differences between genotypes. Individual datapoints, mean and SEM are shown in the dot blots

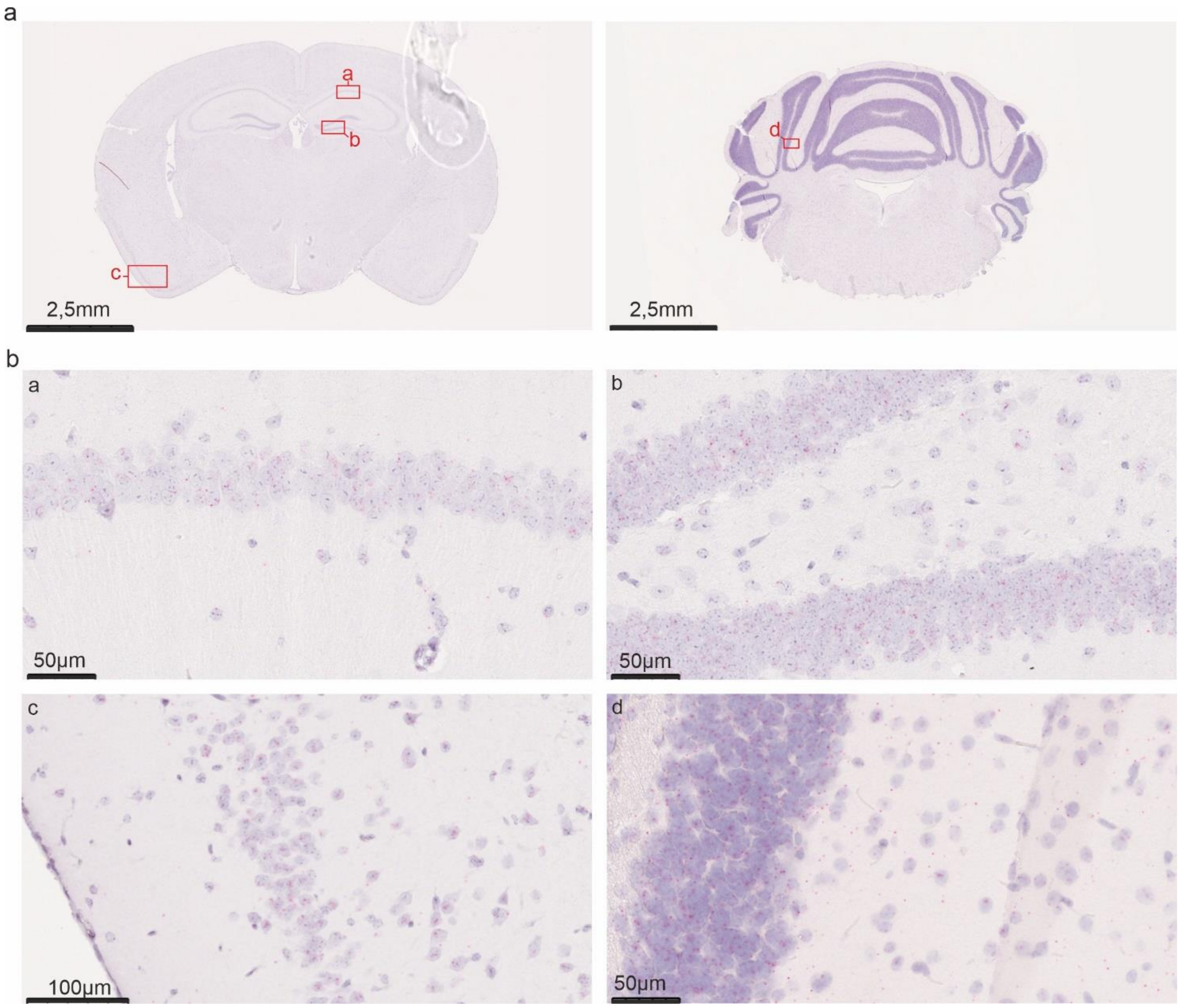

Fig. S11 In situ hybridization of *Nhlrc2* in of the brain of a 32-week-old *Nhlrc2*$^{\text{FINCA/-}}$ male mouse. **a** Overview image of ISH with *Nhlrc2* probe. Red boxes mark the magnified areas: a CA1 of hippocampus, b dentate gyrus of hippocampus, c piriform cortex and d cerebellar hemisphere. **b** *Nhlrc2* expression in areas a‒b show a similar distribution to that in wild-type mouse

SUPPLEMENTAL TABLES

Table S1 Genotyping primers

Primer name	Nucleotide sequence
Nhlrc2_119354_F	AAGGCCCTGTGCTTTCTCTG
Nhlrc2_119354_R	AAGGCTGGCTAGAGAGGACTTG
LacZ_probe_F	GCCCATCTACACCAACGTGA
LacZ_probe_R	CAACCACCGCACGATAGAGA
CRISPR_F (4)	GTGTAGTTTGGAGACATGTGAG
CRISPR_R (5)	CTGTCCCCTGTCTTTGTAATAC

Table S2 qPCR primers

Primer name	Nucleotide sequence
mNhlrc2_F	TGGGATGTTTTCAGAGTCGTCTT
mNhlrc2_R	TCTCAGCTTCCAGGTCTATCTTT
Nhlrc2-f	ACCTTCACCATTGCTGTTCC [22]
Nhlrc2-r	CCTTCCAGGGTTAGGTCCTC [22]
mVcp_F	TGACCCTCATGGATGGCCTA
mVcp_R	TGTCAAAGCGACCAAATCGC
mAnxa6_F	TCATCGACATCGTCACCCAC
mAnxa6_R	AGCCATTAAATCCCGGCCAA
mPls-3_F	CAGCGCTGACATCAAGGATTC
mPls-3_R	TCTTTCTGTCCCTTCGGTGC
mSnx6_F	AGTCGTTCGACAACATGAGGA
mSnx6_F	TGGTGCTGGTGGGATAATGT
mHnrnpc_F	GTGTGAAACGATCTGCAGCG
mHnrnpc_R	TGAGGAACTGAGTAGAGGGGAC
mUbl7_F	GCAGTGACCCCATTGCTCTA
mUbl7_R	GGGCAGGCACCAATGTATCT
mGpd1l_F	TCGGGGAACTGGGGATCAG
mGpd1l_R	CCGTTCACGGTCTCCTCAAA
mTimm29_F	GCAGGGAGACGATGGTGACA
mTimm29_R	GGCCCAGGTGCTCAGTCG
mUba1_F	CGGCAGCTGTACGTTTTGG
mUba1_R	ATTTCTACACCCAAGCCCCG
mPrdx6_F	TGCCATCCTTTTGGGCATGT
mPrdx6_R	GGGCCAAAAATGAACACCACA
mPsma2_F	CGCTGACTACATTCAGCCCA
mPsma2_R	TTTAATCCCCACTGACGGGG
mFtl1_F	CGGACCCTCATCTCTGTGAC

mFtl1_R	AGGTTGGTCAGATGGTTGCC
mRbm8a_F	GACGTGCTGGATCTTCACGA
mRbm8a_R	GTGGATGCTTTCGTCCCCAT
mPfdn2_F	CTACCGCATGGTTGGAGGTG
mPfdn2_R	TCTGTATCTGCTCCTTGTTGCC
mHist1h4a_F	GATTCTGTTGGGGGTGGGAG
mHist1h4a_R	GCGACCCGACATGTCTTAGG
mDynlrb1_F	ACACAGAAGGCATTCCCATCA
mDynlrb1_R	GGGTCAATCTCACGCACAGT
mAbracl_F	GGGTTCCAGAAATGCTGATGGA
mAbracl_R	CAACGCTTCAAAGAGATTGGCA
mPea15_F	ACAGAACCCGTGTGCTGAAG
mPea15_R	GCTGCCGGATAATGTCTTTGTA
mBrk1_F	TCTTGTCGTTCAAGACTCGCA
mBrk1_R	CTCACCCTTTGTCACCCTCG
mRPL13Aq_F	GAGGTCGGGTGGAAGTACCA
mRPL13Aq_R	TGCATCTTGGCCTTTTCCTT
mPGK1q_F	CTGACTTTGGACAAGCTGGACG
mPGK1q_R	GCAGCCTTGATCCTTTGGTTG

Table S3 Genotype distribution of offspring obtained by mating Nhlrc2 $^{FINCA/-}$ male mice with either Nhlrc2 $^{FINCA/-}$ or wild-type Nhlrc2 $^{+/+}$ females

Breeding pairs				Offspring genotypes					
			Pups	Nhlrc2$^{FINCA/-}$		Nhlrc2$^{FINCA/FINCA}$		Nhlrc2$^{-/-}$	
male	female	Pairs	(N)	observed	expected	observed	expected	observed	expected
Nhlrc2 $^{FINCA/-}$	Nhlrc2 $^{FINCA/-}$	2	15	11	7.5	4	3.75	0	3.75
				Nhlrc2$^{+/-}$ observed	expected	Nhlrc2 $^{FINCA/+}$ observed	expected		
Nhlrc2 $^{FINCA/-}$	Nhlrc2 $^{+/+}$	2	10	5	5	5	5		

Table S4 Blood values from Nhlrc2 $^{FINCA/-}$ and wild-type Nhlrc2 $^{+/+}$ mice

	Hb (g/l)	SD	Hkr (%)	SD	splenic wt (g)	SD
Nhlrc2 $^{FINCA/-}$ female (N=6)	141.0	5.19	43.8	1.51	0.09 (N=4)	0.007
Nhlrc2 $^{+/+}$ female (N=5)	138.8	3.97	43.2	1.23	0.09 (N=4)	0.008
Nhlrc2 $^{FINCA/-}$ male (N=6)	138.8	8.05	43.4	1.50	0.087 (N=5)	0.018
Nhlrc2 $^{+/+}$male (N=6)	146.2	5.05	45.5	1.69	0.084 (N=5)	0.011

Table S5 Detailed statistical and MS data about the identified proteins

Spot	Protein	UniProt-KB	Mean normalized spot volume *Nhlrc2* $^{+/+}$	*Nhlrc2* $^{FINCA/-}$	MSMS score	SC (P)
1	VCP	Q01853	244±53	118±100	2236	56(32)
2	ANXA6	P14824	211±62	389±77	1156	51(27)
3	PLS3	Q99K51	202±38	327±86	1119	45(23)
4	SNX6	Q6P8X1	174±37	281±53	1556	38(15)
5	hnRNP C1/C2	Q9Z204	259±71	399±72	1118	40(10)
6	UBL7	Q91W67	299±89	586±155	639	32(7)
7	GPD1L	Q3ULJ0	154±45	237±51	2010	52(14)
8	TIMM29	Q8BGX2	122±36	191±58	406	33(9)
9	UBA1	Q02053	117±49	288±97	496	7(6)
10	PRDX6	O08709	226±17	429±58	1012	60(10)
11	PSMA2	P49722	1464±23	242±33	536	53(8)
12	FTL1	P29391	301±69	534±90	1448	56(7)
13	RBM8A	Q9CWZ3	265±55	426±66	1323	61(6)
14	FTL1	P29391	211±52	451±109	1381	48(9)
15	PFDN2	O70591	222±48	374±79	2145	51(8)
16	HIST1H4A	P62806	249±43	403±87	3203	62(8)
17	HIST1H4A	P62806	108±39	312±87	1417	44(5)
18	DYNLRB1	P62627	207±42	347±59	1698	83(7)
19	ABRACL	Q4KML4	315±72	514±109	839	77(4)
20	PEA15	Q62048	335±128	685±164	844	73(9)
21	BRK1	Q91VR8	268±59	485±114	831	67(5)

UniProt-accession numbers, as well as the mean normalized spot volumes and their corresponding standard deviations, are presented. Parameters of the protein identification, comprising MSMS Mascot scores as well as sequence coverage (SC) and covered peptides (P), are shown

Table S6 qPCR results showing expression levels of genes identified in the 2D-DIGE

Gene	Normalized expression wt (n=3) mean	st. dev	FINCA/KO (n=3) mean	st. dev	Relative quantity	t-Test
Vcp	1.08	0.14	1.05	0.13		
Anxa6	0.91	0.09	0.68	0.03	0.75	0.023
Pls3	1.17	0.13	1.02	0.06		
Snx6	0.94	0.05	0.98	0.05		
Hnrnpc	0.92	0.07	0.64	0.05	0.70	0.012
Ubl7	0.95	0.06	1.05	0.12		
Gpd1l	0.73	0.20	0.71	0.12		

Timm29	1.19	0.19	1.27	0.08		
Uba1	0.88	0.11	0.92	0.03		
Prdx6	1.02	0.04	0.82	0.12		
Psma2	1.09	0.09	1.24	0.32		
Ftl1	1.05	0.05	0.84	0.08	0.80	0.035
Rbm8a	1.23	0.18	0.98	0.05		
Pfdn2	0.80	0.14	0.79	0.07		
Hist1h4a	1.21	0.17	0.94	0.28		
Dynlrb1	0.97	0.02	0.94	0.10		
Abracl	0.96	0.10	1.12	0.09		
Pea15	0.84	0.11	0.80	0.07		
Brk1	0.98	0.06	0.99	0.07		
