# Supplementary material for: Variant in NHLRC2 leads to increased hnRNP C2 in developing neurons and the hippocampus of a mouse model of FINCA disease
Source: Mol Med. 2020 Dec 9;26:123. doi: 10.1186/s10020-020-00245-4 (PMC7724728; doi:10.1186/s10020-020-00245-4)
Supplement: Supplementary file 2 — Additional file 2: Unedited full images of the immunoblots for Fig. 2, Fig. 4, Fig. S2, Fig. S3, Fig S8, and Fig S10. [file 10020_2020_245_MOESM2_ESM.pdf]

Variant in NHLRC2 leads to increased hnRNP C2 in developing neurons and the hippocampus of a mouse model of FINCA disease

Anniina E. Hiltunen [1, 2], Salla M. Kangas [1, 2], Steffen Ohlmeier [3], Ilkka Pietilä [1,4], Jori Hiltunen [1], Heikki Tanila [5], Colin McKerlie [6, 7], Subashika Govindan [8], Hannu Tuominen [9, 10], Riitta Kaarteenaho [11, 12], Mikko Hallman [1], Johanna Uusimaa [1, 13], Reetta Hinttala [1, 2].

[1] Medical Research Center Oulu and PEDEGO research unit, University of Oulu and Oulu University Hospital, Oulu, Finland

[2] Biocenter Oulu, University of Oulu, Oulu, Finland

[3] Proteomics Core Facility, Biocenter Oulu, Faculty of Biochemistry and Molecular Medicine, University of Oulu, Oulu, Finland

[4] Department of Immunology, Genetics and Pathology, Science for Life Laboratory, Uppsala University, Rudbeck Laboratory, Uppsala, Sweden

[5] A.I. Virtanen Institute, University of Eastern Finland, Kuopio, Finland

[6] The Hospital for Sick Children, Toronto, Canada

[7] Faculty of Medicine, University of Toronto, Toronto, Canada

[8] Tissue Engineering Laboratory, Hepia/HES-SO, University of Applied Sciences Western Switzerland, Geneva, Switzerland

[9] Department of Pathology, Cancer and Translational Medicine Research Unit, University of Oulu, Oulu, Finland

[10] Department of Pathology, Oulu University Hospital, Oulu, Finland

[11] Research Unit of Internal Medicine, Respiratory Research, University of Oulu, Oulu, Finland

[12] Medical Research Center Oulu and Unit of Internal Medicine and Respiratory Medicine, Oulu University Hospital, Oulu, Finland

[13] Clinic for Children and Adolescents, Paediatric Neurology Unit, Oulu University Hospital, Oulu, Finland

Corresponding author:
Anniina E. Hiltunen, M.Sc.
PO Box 5000
FIN-90014 University of Oulu

Email: anniina.hiltunen@oulu.fi

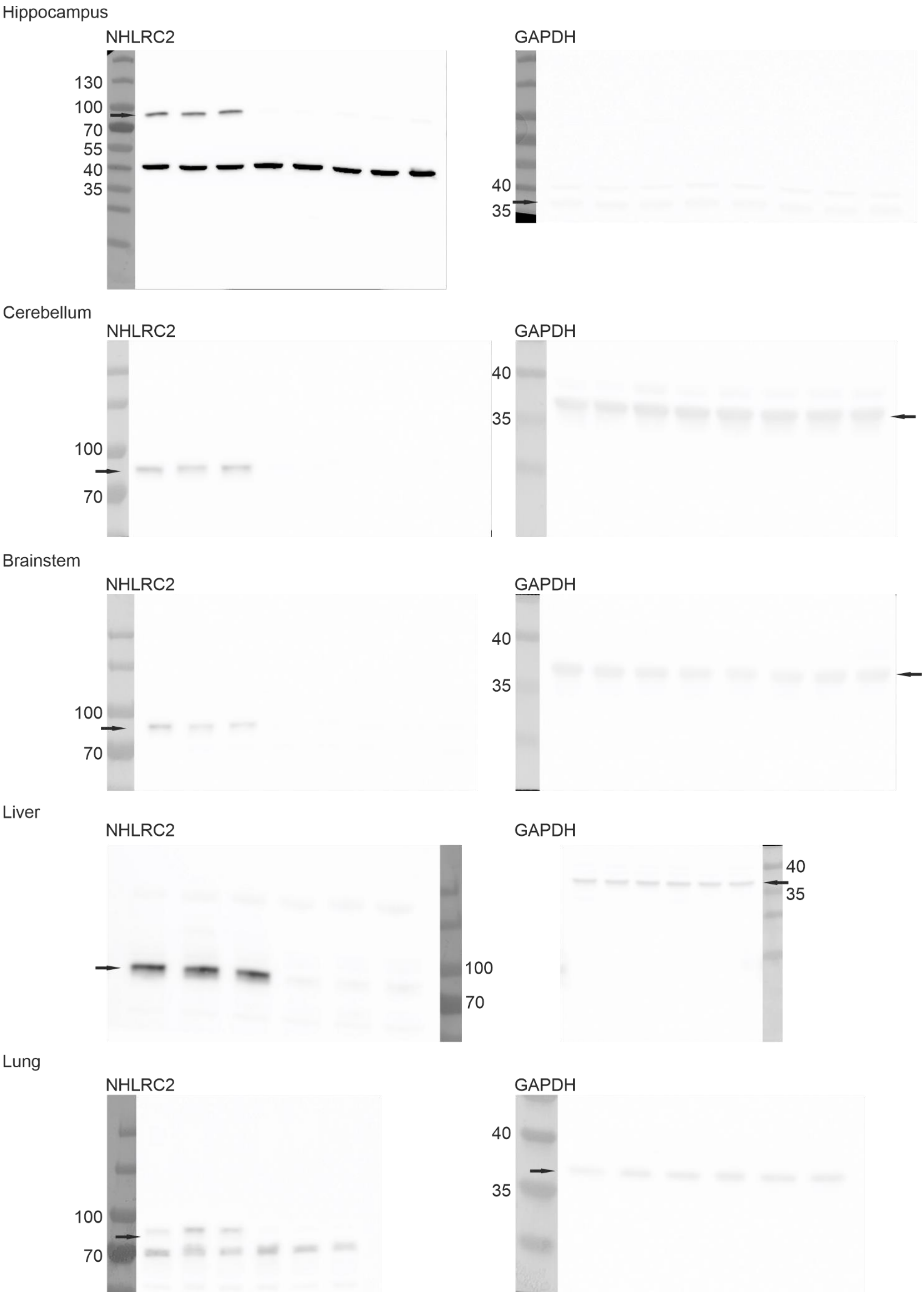

Full unedited gels for Figure 2

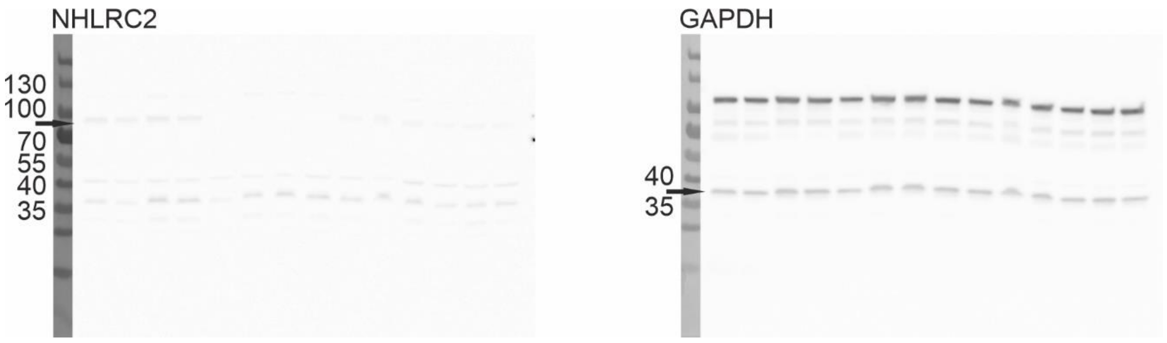

Full unedited gels for Figure 4

Fig 5b

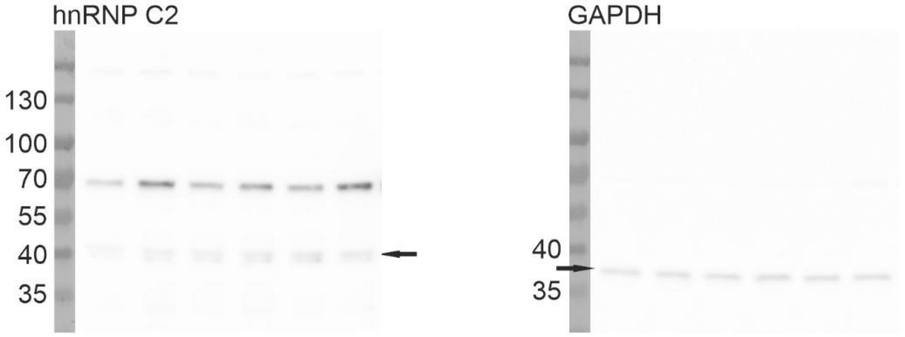

Fig 5c

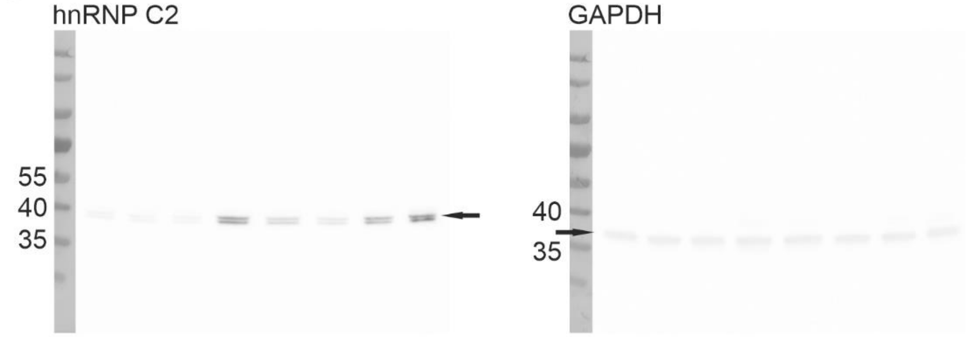

Fig 5d

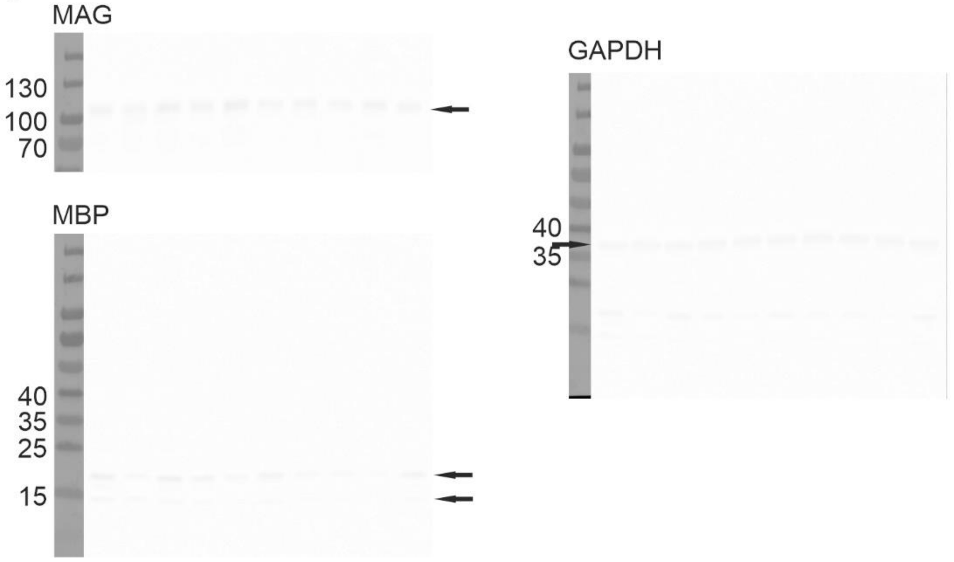

Full unedited gels for Figure 5

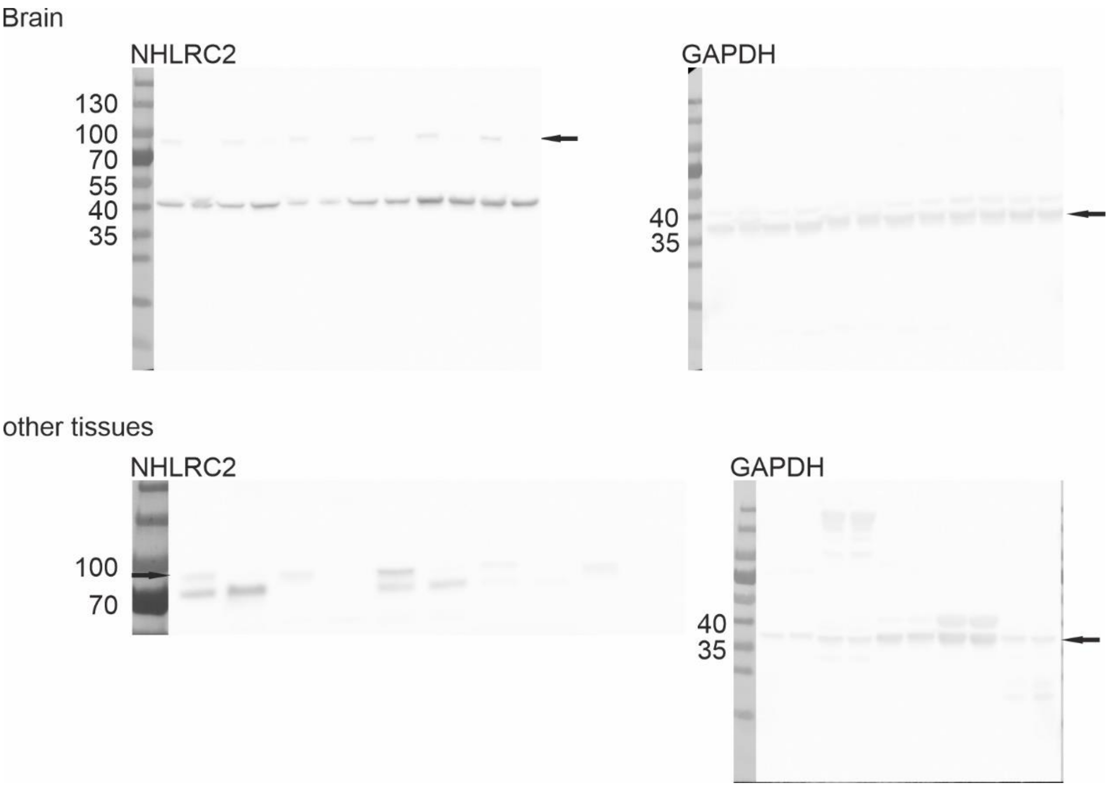

Full unedited gels for Figure S2

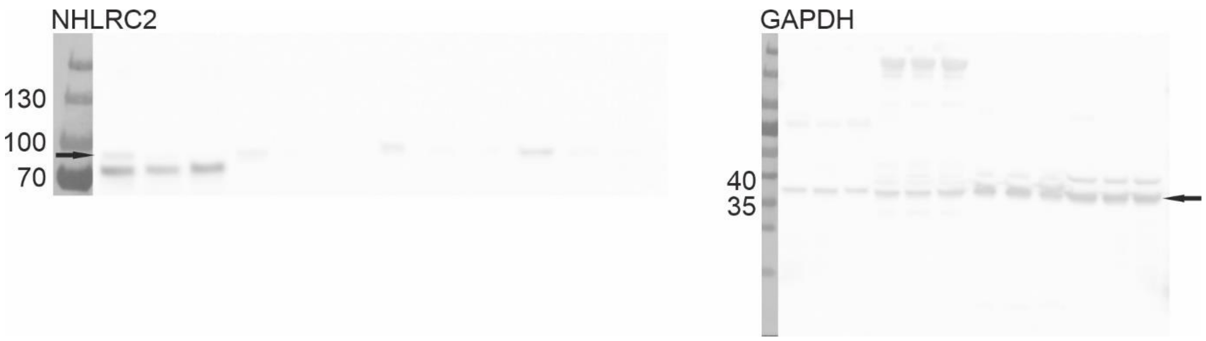

Full unedited gels for Figure S3

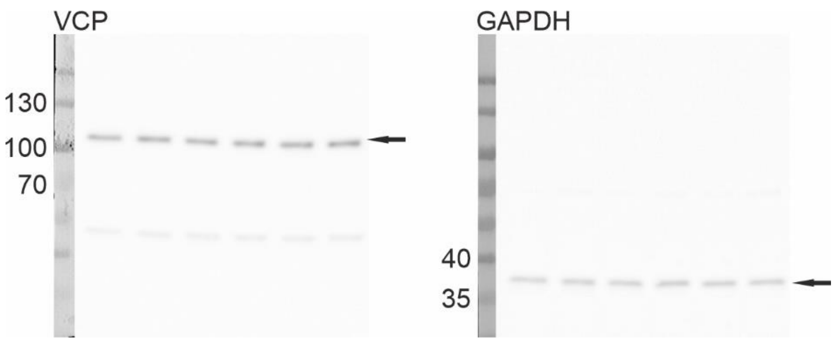

Full unedited gels for Figure S8

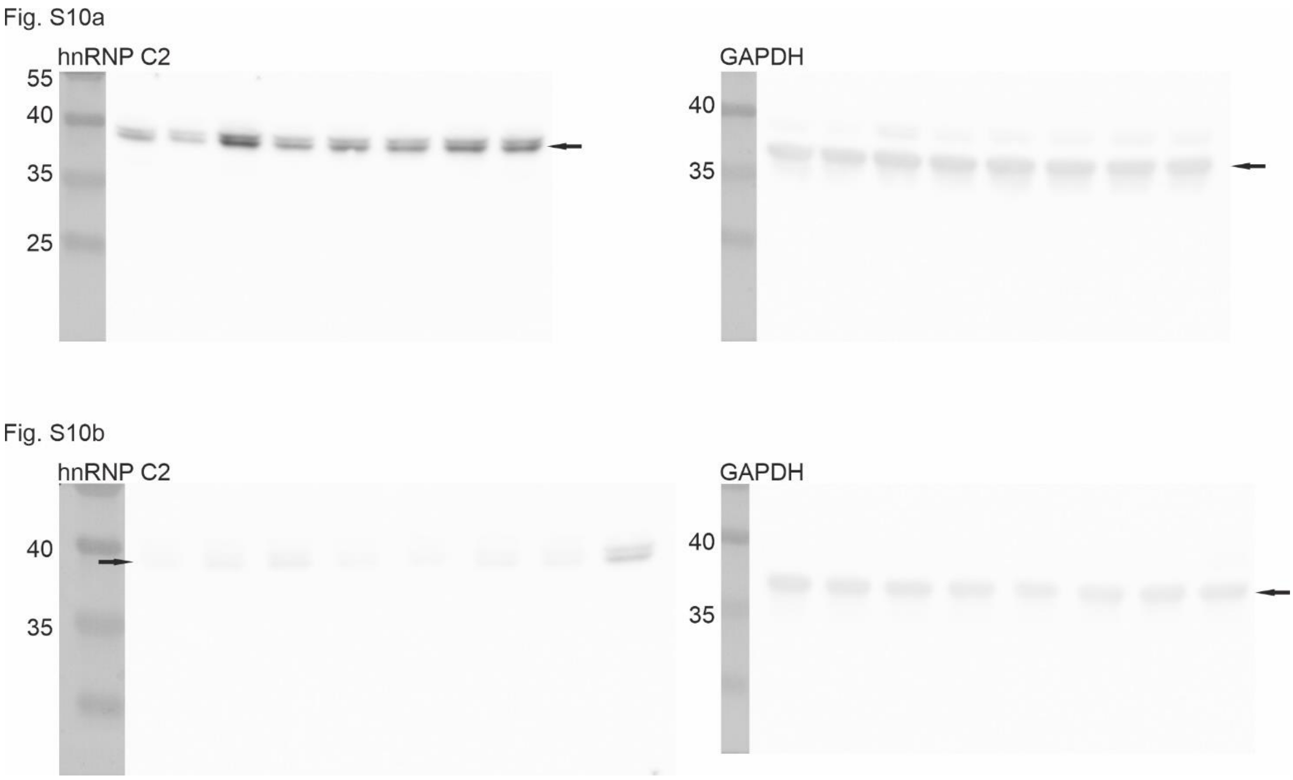

Full unedited gels for Figure S10
